# A novel algorithm based on ensemble empirical mode decomposition for non-invasive fetal ECG extraction

**DOI:** 10.1371/journal.pone.0256154

**Published:** 2021-08-13

**Authors:** Katerina Barnova, Radek Martinek, Rene Jaros, Radana Kahankova, Adam Matonia, Michal Jezewski, Robert Czabanski, Krzysztof Horoba, Janusz Jezewski

**Affiliations:** 1 Department of Cybernetics and Biomedical Engineering, Faculty of Electrical Engineering and Computer Science, VSB–Technical University of Ostrava, Ostrava, Czechia; 2 Łukasiewicz Research Network –Institute of Medical Technology and Equipment, Zabrze, Poland; 3 Department of Cybernetics, Nanotechnology and Data Processing, Silesian University of Technology, Gliwice, Poland; Effat University, SAUDI ARABIA

## Abstract

Non-invasive fetal electrocardiography appears to be one of the most promising fetal monitoring techniques during pregnancy and delivery nowadays. This method is based on recording electrical potentials produced by the fetal heart from the surface of the maternal abdomen. Unfortunately, in addition to the useful fetal electrocardiographic signal, there are other interference signals in the abdominal recording that need to be filtered. The biggest challenge in designing filtration methods is the suppression of the maternal electrocardiographic signal. This study focuses on the extraction of fetal electrocardiographic signal from abdominal recordings using a combination of independent component analysis, recursive least squares, and ensemble empirical mode decomposition. The method was tested on two databases, the Fetal Electrocardiograms, Direct and Abdominal with Reference Heartbeats Annotations and the PhysioNet Challenge 2013 database. The evaluation was performed by the assessment of the accuracy of fetal QRS complexes detection and the quality of fetal heart rate determination. The effectiveness of the method was measured by means of the statistical parameters as accuracy, sensitivity, positive predictive value, and F1-score. Using the proposed method, when testing on the Fetal Electrocardiograms, Direct and Abdominal with Reference Heartbeats Annotations database, accuracy higher than 80% was achieved for 11 out of 12 recordings with an average value of accuracy 92.75% [95% confidence interval: 91.19–93.88%], sensitivity 95.09% [95% confidence interval: 93.68–96.03%], positive predictive value 96.36% [95% confidence interval: 95.05–97.17%] and F1-score 95.69% [95% confidence interval: 94.83–96.35%]. When testing on the Physionet Challenge 2013 database, accuracy higher than 80% was achieved for 17 out of 25 recordings with an average value of accuracy 78.24% [95% confidence interval: 73.44–81.85%], sensitivity 81.79% [95% confidence interval: 76.59–85.43%], positive predictive value 87.16% [95% confidence interval: 81.95–90.35%] and F1-score 84.08% [95% confidence interval: 80.75–86.64%]. Moreover, the non-invasive ST segment analysis was carried out on the records from the Fetal Electrocardiograms, Direct and Abdominal with Reference Heartbeats Annotations database and achieved high accuracy in 7 from in total of 12 records (mean values *μ* < 0.1 and values of ±1.96*σ* < 0.1).

## Introduction

Fetal monitoring is an integral part of modern maternal and fetal care, used mainly to prevent fetal hypoxia. Hypoxia is a life-threatening condition characterized by insufficient oxygen supply to the body or individual tissues. If hypoxia is diagnosed, it is necessary to terminate the pregnancy by caesarean section as soon as possible [1]. Currently, several methods for monitoring fetal cardiac activity are known. The oldest technique is listening to heart sounds with a stethoscope [2]. Newer monitoring methods include, for example, a non-invasive, low-cost, and completely passive method called phonocardiography. This method is based on recording acoustic heart sounds, vibrations and murmurs of the fetus using an acoustic sensor located on the maternal abdomen [3, 4]. Magnetocardiography based on the measurement of magnetic fields generated by electric currents of the fetal heart is an alternative method. Magnetic fields are detected using highly sensitive superconducting quantum interference device sensors. The advantage of this method is the quality of the signals due to absence of biological noise. Unfortunately, this is a very expensive method requiring a shielded room [5, 6].

Therefore, in clinical practice, a very simple and fast cardiotocography (CTG) method, based on the principle of Doppler ultrasonography, is used for fetal monitoring. This method allows continuous monitoring of fetal heart rate (fHR) and uterine contractions [7, 8]. The first commercially available cardiotocograph model was introduced in 1968 [2]. Since then, there has been a marked decrease in the number of fetal deaths during childbirth, but, subsequently, there has also been an increase in the number of caesarean sections performed [9]. This phenomenon is attributed to the poor reliability and accuracy of this method [10–13]. For example, studies [14, 15] point to this problem and the research [16] confirms the hypothesis that, with the development of fetal monitoring using CTG, the number of caesarean births has increased. An acute caesarean section is a very invasive surgical procedure and, compared to the natural mode of delivery, is thus a great burden for both the mother and the fetus [17]. Hence, although CTG is beneficial in fetal monitoring, this method appears to be insufficient to accurately diagnose the risk of hypoxia.

Invasive monitoring by means of fetal electrocardiography (fECG) is currently used to increase the accuracy of the fetal diagnosis. Invasive monitoring is based on recording the electrical potentials of the fetal heart using a scalp electrode, which is applied to the fetal head [18]. This method also allows, in addition to fHR determination, a morphological analysis of the fECG signal (e.g. to determine the T/QRS ratio also known as ST analysis), which can improve hypoxia detection. The advantage of invasive fECG, compared to its non-invasive version, is the acquisition of a signal containing a low level of noise, which provides accurate information about the health of the fetus. However, the disadvantages are the possibility of performing it only during delivery and the risk of developing infection [14, 18]. For these reasons, many authors are currently focusing on improving the non-invasive version of fECG.

Non-invasive fECG appears to be the most promising technique that could become a commonly used alternative to CTG in clinical practice [19]. In the case of non-invasive fECG monitoring, the electrical potentials of the fetal heart are recorded from the surface of the mother’s abdominal wall using transabdominal electrodes [20, 21]. A non-invasive fECG is then safer compared to the invasive version and can be used during pregnancy and delivery. The disadvantage of non-invasive monitoring is the difficult detection of fQRS complexes, because, in addition to the useful signal, interferences are recorded at the same time, including the maternal ECG signal (mECG) [22]. Moreover, the maternal and fetal components overlap in both the time and frequency domains, and the choice of the appropriate filtration technique and its setting is a challenging task [23].

## State-of-the-art methods for fECG signal extraction

Many of the filtration techniques proposed so far for the purpose of fECG extraction were summarized in the studies [3, 5, 24]. The most commonly used methods, [25–27], benefit from independent component analysis (ICA) that decomposes the input signal into statistically independent source components. Also, principal component analysis (PCA), based on the principle of finding the most statistically significant components in the input signal, was applied [27, 28]. The wavelet transform (WT) method that enables to obtain a time-frequency description of the signal was used to extract fECG in [29, 30]. In [31, 32], the empirical mode decomposition (EMD) method, which decomposes the signal into intrinsic mode functions, was applied. The least mean squares and recursive least squares (RLS) algorithms, based on minimization and calculation of the error function between the desired output and the real output of the adaptive algorithm, were also investigated [1, 33]. Studies [34, 35] presented an application of the adaptive neuro-fuzzy interference system (ANFIS) that uses a combination of fuzzy logic and neural network theory. Finally, a prediction-estimation method based on Kalman filtering, which aims to estimate the waveform of the signal using the present input values and the previously calculated state, was proposed in [36].

Recent studies [31, 32, 37–45] show that the use of a single algorithm is not as promising as a combination of multiple algorithms. The research efforts are, therefore, aimed at testing various combinations of two or more algorithms that would overcome the limitations of individual methods while combining their advantages. Combinations of the methods presented in previous studies will be summarized in the following subsections.

### Single channel methods

The study [31] presented a combination of the EMD and correlation analysis. The main idea was to decompose the abdominal ECG (aECG) signal into individual oscillatory functions using the EMD and then determine the correlation between the reference fECG signal and the oscillatory functions of the aECG signal. Subsequently, the oscillatory functions that correlated with the reference signal the most, were found. The sum of the oscillatory functions provided the enhanced fECG. The authors tested the method on real data and achieved an average accuracy of 100% when detecting the fQRS complexes.

A single channel method combining singular value decomposition and the ICA method was tested in [37]. The objective was to project the input signal onto higher dimensions and, thanks to the statistical independence between the source signals, separate them from each other in order to estimate the fECG. When testing on synthetic data, they did not perform any statistical evaluation, but based on visual comparison, they stated that the algorithm achieved effective fECG extraction.

A combination of singular value decomposition and polynomial classifiers was introduced in [38]. The mECG signal was estimated using the singular value decomposition and subsequently mECG and aECG signals were fed to the input of the polynomial classifiers. The fECG signal was extracted using the dynamics and nonlinearities of the estimated mECG signal. The method was tested on real and synthetic data. They evaluated the extraction quality of synthetic data using the signal-to-noise ratio (SNR) parameter and real data by means of visual comparison. The method was evaluated as suitable for single-channel fECG extraction.

The authors of the study [39] tested a combination of the extended Kalman smoother (EKS) and ANFIS. In the first step, the maternal component was estimated from the aECG signal using the EKS. Subsequently, the estimated component was centred with the aECG signal using the ANFIS. The fECG signal was extracted based on subtracting the estimated mECG from the aECG. The evaluation of filtration efficiency was performed using SNR in synthetic records whereas in real records, it was evaluated by the accuracy of fQRS detection achieving an accuracy of 90.20%.

### Multichannel methods

The multichannel method combining ICA, ensemble empirical mode decomposition (EEMD) and wavelet shrinkage (WS), was tested in [32]. In the first step, the ICA method, which separated the fECG signal from the aECG signals, was applied. Since the fECG signal still contained residues of the mECG component, it had to be further filtered using the EEMD method. In the last step, the residual high-frequency noise was removed using the WS method. The authors evaluated the effectiveness of the method using SNR and mean squared error (MSE) and stated that the limitation of the method was its low computational speed and the fact that it causes changes in signal morphology.

Another multichannel method, based on a combination of periodic component analysis and generalized eigenvalue decomposition was tested in the study [40]. The detection of R-peaks was based on the idea of time-varying periodicity. The cross-correlation between time-delayed samples in different channels represented by a constant time variable was replaced with a calculated phase variable. The method was tested on real aECG recordings. No statistical results were presented in the study, but the proposed algorithm was, according to the authors, effective and time-efficient in fECG extraction.

The combination of ICA and WT was presented in the study [41]. The method is based on the principle of separating the fECG component from the aECG signals using the ICA and the subsequent removal of the residual noise in the fECG signal using WT. The evaluation of the results was performed by visual comparison and authors stated that in addition to the efficient extraction of fECG, the morphology of the signal was preserved.

In the study [42], the FastICA and the quality index optimization (QIO) methods were combined. First, the FastICA, which decomposed the signal into individual components, was applied. Since the fECG contained residues of the mECG signal, the mECG component was subtracted from the fECG. In the last step, the quality of the fECG signal was improved using the QIO method. When evaluating the accuracy of fQRS detection using the F1-score, an accuracy of 99.38% was achieved when tested on real records and an accuracy of 98.78% when tested on synthetic records.

The authors of the study [43] dealt with a machine learning approach integrating an echo state network (ESN) with dynamic programming. The idea was based on the detection of fQRS complexes using the recurrent ESN. The resulting positions of the fQRS complexes were obtained by dynamic programming interpreting the outputs of the ESN. The algorithm was tested on real records, in which it was able to effectively suppress the maternal component and the extraction of fECG was thus efficient. The method was relatively fast, but further improvement should be possible by increasing the ESN contribution.

An artificial neural network and correlation-based technique were combined in study [44]. First, the mECG component was extracted from the aECG signals using the artificial neural network. Based on the correlation, the mECG component was centred with the aECG signals. Finally, the mECG component was subtracted from the aECG signals, and the fECG signal was obtained. The method was tested on real records and an accuracy of 93.75% was achieved in the detection of fQRS complexes.

Finally, a combination of correlation analysis and the FastICA was presented in [45]. First, the self-correlation analysis was applied to reduce the temporal correlation. Subsequently, the FastICA method was applied, which decomposed the input signal into individual components, one of which was the resulting fECG signal. The authors did not use any statistical parameters to evaluate the extraction efficacy, but according to the visual evaluation, the extracted fECG signals contained almost no maternal residues.

This work is based on the methods we proposed in our previous studies. In [46], we presented the ICA-ANFIS-WT and ICA-RLS-WT procedures and, in [47], we tested the ICA-EMD, ICA-EMD-WT and ICA-RLS-EMD approaches. When determining the fHR, all methods provided better results than using its individual components separately. However, the disadvantage of the methods, which includes the WT, was the deformation of the fQRS complexes, which would not allow the subsequent morphological analysis of the signal, such as the ST analysis. The goal of this study is to introduce an algorithm composed of ICA, RLS and EEMD methods, which could provide better results in determining the fHR than the previously presented approaches, it would be effective in a wide range of signals and would allow the subsequent morphological analysis of the fECG.

### ST segment analysis

In addition to fHR, which is the main parameter assessing the fetal well-being, new techniques focusing on the changes of the fECG waveform (P-R interval, QT interval, T/QRS ratio and ST segment) caused by the hypoxic states have been developed and presented in the literature [41, 48–55]. However, the STAN^®^ Method (Neoventa Medical, Sweden) is the only analysis tool that is available in the clinical practice. The combination of ST-Analysis provided by the STAN^®^ device and standard CTG parameters (the trace of both fHR and uterine contractions) provides extended and more accurate assessment of fetal well-being during the labor than CTG alone.

**Fig 1:**
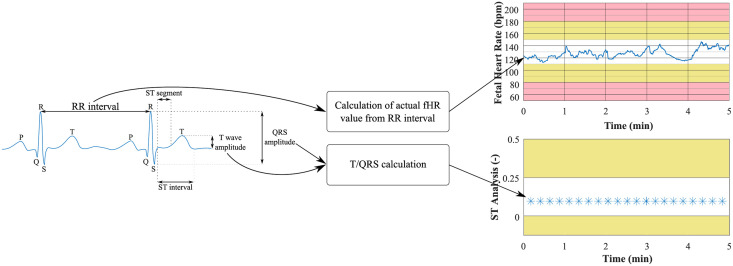
Principle of fHR calculation and determination of ST analysis inspired by STAN devices.

*The principle*: The analysis of the fECG using STAN^®^ is possible only during the labor (after membrane rupture) since it requires the scalp electrode to be placed on the fetal body. The STAN device analyses 30 consecutive ECG complexes and compares them with the ‘baseline value’, which was calculated over the first four to five minutes for each fetus. Each analysis is marked by ‘X’ on the lower part of the monitor (see Fig 1). In the presence of an ‘ST event’ (value differing significantly from the “baseline value”), it is necessary to classify the CTG trace according to STAN guidelines and then to determine if any action is needed [56].

## Material and methods

This section describes the component methods that were selected based on the literature review and the results of our previous research. Within the system, the following methods were implemented: ICA, RLS and EEMD. The databases on which the algorithm was tested, and the description of statistical parameters used to determine the effectiveness of the filtration method in fQRS complexes detection, are also described.

### Independent component analysis

Independent component analysis is a statistical method based on separation of mixed independent signals and extraction of the original source components [57]. In order to provide good results, when using ICA, two assumptions must be met: the source signals must be independent, and only one original component can have a Gaussian distribution. If the method is applied to signals where there are multiple Gaussian sources, then it cannot extract these sources [27, 58]. In recent years, many improved ICA algorithms have been proposed [26, 59–62]. One of them, the FastICA algorithm, is widely used due to its fast convergence. FastICA is a fixed-point iterative algorithm, minimizing mutual information between the estimated components. There are various types of FastICA algorithms, including those based on kurtosis, maximum likelihood, and maximum negentropy [25]. A detailed mathematical description of the method can be found in studies [25, 27, 57, 58, 63].

### Recursive least squares

Recursive least squares is an adaptive method based on minimizing the error defined as the difference between the desired output and the real output of the adaptive algorithm. The minimization of the error function is achieved by automatic adjustment of the adaptive filter coefficients [1]. The RLS algorithm is remarkable due to its fast convergence and provides excellent results in time-varying environments [64]. Nevertheless, in its basic form it has higher computational demands and, in some cases, it may have stability problems [1]. To reduce the computational demands, the RLS algorithm uses “adaptation” or “forgetting” factor λ, whose task is to decrease the contribution of “old” error values. The forgetting factor value ranges from zero to one [1, 64]. To reduce the computational complexity, a filter order which indicates the final number of the previous values processed, is also selected. Proper setting of the RLS algorithm parameters has a great effect on the stability of the filter [1]. Further information about RLS can be found in studies [1, 64–66].

### Ensemble empirical mode decomposition

The ensemble empirical mode decomposition is an improved version of the empirical mode decomposition (EMD) method. The EMD is based on the decomposition of the non-stationary signal, such as the biomedical signal, into oscillatory functions called intrinsic mode functions (IMFs) [67]. The EMD algorithm works on the principle of identifying local extremes of the signal and creating the upper and lower envelopes of the signal. The mean of these envelopes is subtracted from the input signal, thus obtaining the first IMF. The entire process is repeated, but instead of the input signal, the residual signal which was obtained by subtracting the IMF from the input signal, is used. The iterative process terminates when the final residual signal is extracted [47, 68]. A more detailed description of the EMD method can be obtained in studies [31, 32, 47, 67].

**Fig 2:**
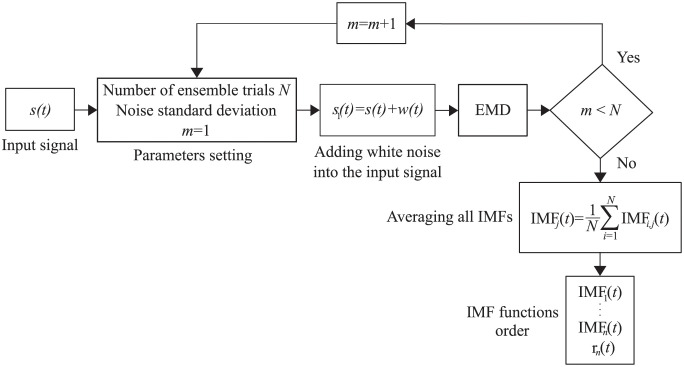
Block diagram of the EEMD. The method works on the principle of adding white noise into the input signal and performing *N* ensemble trials. The final IMFs are obtained by averaging the results of these trials, the *r*_*n*_(*t*) is a residual signal.

Despite many advantages, the EMD has a limitation called *mode mixing*, which can significantly affect the decomposition efficiency. This phenomenon occurs when one of the IMFs contains several components with very different frequencies, or when components with very similar frequencies are contained in different IMFs [32]. To prevent this phenomenon, EEMD was proposed in the study [69], based on adding different series of white noise into the input signal in several trials [70]. Further information and a more detailed description of this method can be found in studies [32, 67, 69–71]. A brief description of the method can be summarized in the following steps:

1. Set the number of ensemble trials *N* and the standard deviation of the added noise series *N*_*std*_.
2. Add a white noise series *w*(*t*) into the input signal *s*(*t*):
  $$\begin{array}{c} s_{1}\left(t\right)=s\left(t\right)+w\left(t\right). \end{array}$$ (1)
3. Decompose *s*_1_(*t*) signal using the EMD algorithm.
4. Repeat step 1. and 2. *N*-times, but with different white noise series each time.
5. The final *IMF*_*j*_(*t*) of the EEMD is obtained by averaging all IMFs related to *N* trials:
  $$\begin{array}{c} IMF_{j}\left(t\right)=\frac{1}{N}\sum_{i=1}^{N}IMF_{i,j}\left(t\right), \end{array}$$ (2)
  where *i* is a trial number and *j* is the IMF scale. The principle of the EEMD algorithm is shown in Fig 2.

These methods were selected for following reasons:

1. ICA—the ICA method is a separation technique able to decompose a composite input signal, such as aECG, into its components, such as fECG, mECG, and noise [46, 47, 72]. The advantage of the FastICA variant is that it requires little memory space, is conceptually simple and computationally efficient, as it uses a fixed iteration scheme [25, 73]. Previous studies [27, 46, 47, 74] have shown that when processing fECG, the ICA method could highlight the fetal component in the aECG signal, but the maternal component was not sufficiently eliminated. On the other hand, ICA was able to extract the mECG signal very accurately. The advantage of the ICA application in fECG processing is that it requires only signals from the abdominal electrodes as input and there is no need to record the reference mECG signal by means of the chest electrodes [24]. This brings benefits, such as greater comfort and mobility, especially for the mother. Compared to the PCA method, which is also often used to estimate the maternal component, ICA is able to obtain a more accurate estimate, and in addition, ICA produces both maternal component (mECG signal) and an aECG signal with an enhanced fetal component [27].
2. RLS—the advantage of the RLS algorithm is its adaptability and high performance in time-varying environments which makes it suitable for processing fECG [5, 75]. However, its performance is highly dependent on the quality of the input signals. When high quality aECG and mECG signals enter the algorithm, RLS usually achieves very promising results in fECG extraction. There are two different approaches to achieve the fECG extraction. The first approach uses an abdominal signal as a primary input, whereas the signal is recorded using the chest electrode as reference. The second approach is based on using solely abdominal recordings and an algorithm that is able to separate the maternal reference from an aECG signal [5]. When using only the latter approach, the obtained mECG reference is more similar to the maternal component in corresponding aECG signal, and thus the adaptive system can achieve better results. The thoracic signal may be of poor quality, its continual measurement is uncomfortable for the mother, and change in the sensor location leads to changes in the resulting signal’s morphology, which may differ from the maternal component than in the abdominal area and extraction by the RLS algorithm is thus more difficult and less effective [1, 5]. Frequently used adaptive algorithms also include the least mean squares methods, however, the experimental results show that the RLS algorithm outperforms it [1, 76], although at the cost of higher computation time.
3. EEMD—the EEMD algorithm is suitable for processing non-stationary and non-linear signals, such as fECG [32, 67]. In most studies [32, 47, 77–79] that deal with the extraction of fECG using EMD-based methods, these methods are used in combination with other methods, as they are not able to extract either fECG or mECG precisely enough. However, EMD-based methods excel in the final smoothing of the residuals of the maternal component in the fECG signal, which was previously extracted by another more efficient method such as adaptive RLS. In addition, the methods allow the subsequent morphological analysis of the resulting signal to be performed. This makes them suitable as part of diagnostic systems that, in addition to accurately determining fHR, require a more detailed morphological analysis of the fECG waveform [47]. Compared to the EMD method, which is also very often used for the final smoothing of the fECG signal, EEMD is more efficient because it is less limited by the mode mixing problem. However, the disadvantage of EEMD is its lower computing speed [80, 81].

To achieve accurate results, this study proposed a combination of these three methods, by incorporating them in a way that their aforementioned benefits are utilized:

- ICA alone is not able to estimate fECG accurately enough, but it can excellently estimate mECG and aECG signal with an enhanced fetal component. At the same time, the ICA generates input signals for the RLS method, using solely aECG signals.
- As the RLS is prone to poorly scanned reference mECG signals, using high-quality input signals estimated by the ICA method, the RLS can extract high-quality fECG with minor maternal residues.
- These maternal residues are eventually smoothed by the EEMD method, which as such is not able to completely extract either fECG or mECG. In addition, it allows the subsequent morphological analysis of the resulting signal to be performed.

## Dataset

The study protocol was approved by the Ethical Committee of the Silesian Medical University, Katowice, Poland (NN-013–345/02). Subjects read the approved consent form and gave written informed consent to participate in the study.

In this study, two databases were used to test the performance of the ICA-RLS-EEMD extraction method. The first one is called the *Fetal Electrocardiograms, Direct and Abdominal with Reference Heartbeats Annotations* (FECGDARHA) and is available in the *figshare repository* [82]. It contains 12 real recordings acquired from 12 different women, in 38–42 week of pregnancy. The recordings contain four abdominal signals and one signal from the scalp electrode forming the reference. The signals of five minutes length each were digitized with a 16-bit resolution at 500 Hz sampling frequency for abdominal signals and 1000 Hz for a direct reference fECG [83]. The recordings were registered using the KOMPOREL system. This system automatically detected and marked R-peaks, whose accuracy was verified offline by a group of cardiologists, providing the reference annotations [82, 83]. A detailed description of the database can be found in [83].

The second dataset considered is the *Physionet Challenge 2013 database* [84], from which we analysed the first 25 out of 75 recordings. This database was created for the *Computing in Cardiology Challenge 2013* in order to improve the detection of fQRS complexes and the determination of the fQT interval from a non-invasive abdominal recording [85]. Each recording contains four abdominal signals with a sampling frequency of 1000 Hz and annotations indicating the positions of R-peaks. The length of each recording is one minute. The recordings come from different sources and are acquired using a variety of devices with different resolutions and configurations. The fetuses do not have the same gestational age and no attention was paid to the placement of the electrodes [84].

## Extraction system design

**Fig 3:**
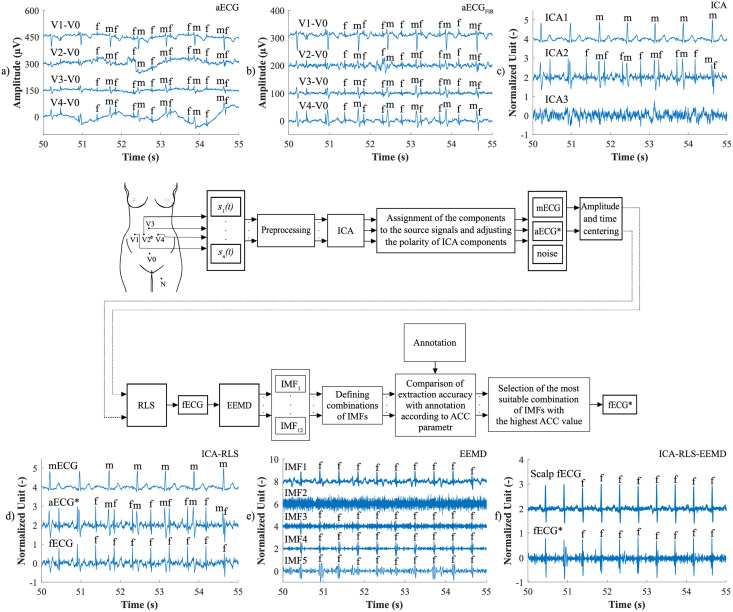
Block diagram explaining the ICA-RLS-EEMD method: a) input aECG signals; b) input aECG_FIR_ signals preprocessed by the FIR filter; c) three source components extracted by the ICA method; d) ICA components assigned to the source signals, which were time and amplitude centred and served as inputs to the RLS algorithm; the fECG signal was the output of the ICA-RLS algorithm; e) the first five IMFs that were obtained after the application of the EEMD method; f) a reference scalp electrode recording and the resulting fECG*, which was extracted after applying the ICA-RLS-EEMD method.

The ICA-RLS-EEMD algorithm was based on several steps. The first was the decomposition of input aECG signals into three components using the ICA. However, only two components were used for further processing. The first was the mECG component and the second was the component in which the mECG signal was most suppressed and the fECG signal was most enhanced. These two signals were used as the inputs of the adaptive RLS system. The RLS output comprised an fECG signal which was still corrupted with residual noise. Therefore, the EEMD method was applied in order to remove the noise and obtain an enhanced fECG*. The block diagram of the algorithm and the output signals are shown in Fig 3.

**Fig 4:**
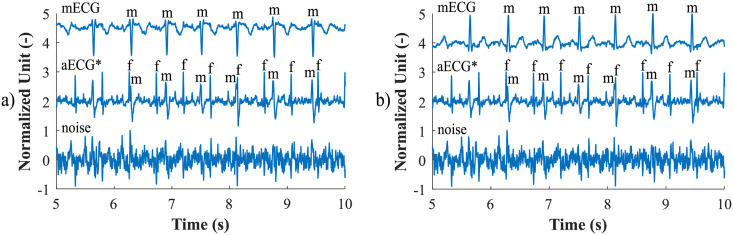
Decomposition of the input aECG signal using the ICA method on three output components (mECG, aECG* and noise): a) inverted polarity of the mECG component; b) mECG polarity correction using the proposed algorithm.

**Fig 5:**
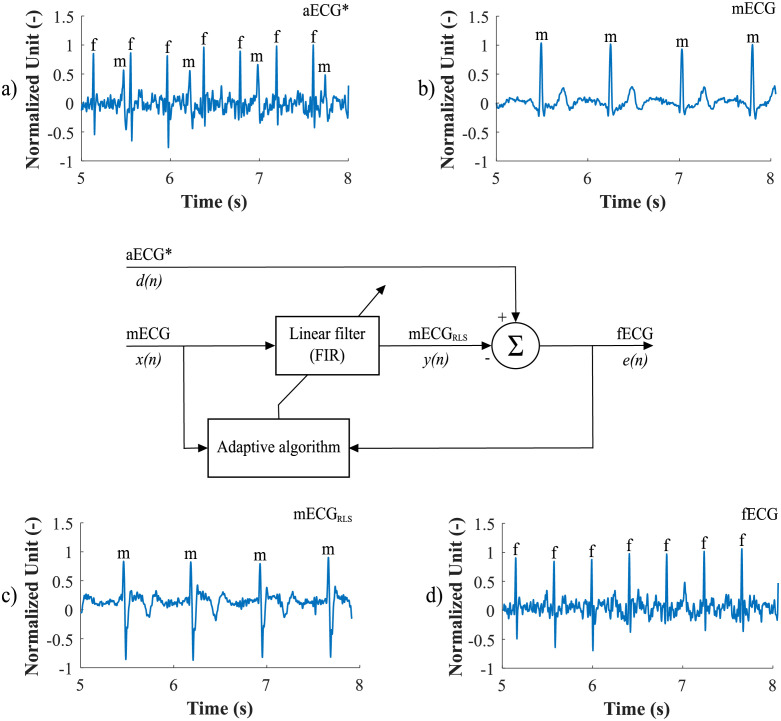
The RLS algorithm structure with examples of the input and output signals: a) aECG* signal, referred to as primary input or desired signal *d*(*n*), b) mECG signal that needed to be adjusted by an adaptive filter, denoted as *x*(*n*). Example c) represents an mECG_RLS_ component that has been adjusted by the filter into a shape of the mECG component in the aECG* signal, denoted as *y*(*n*). This modified mECG_RLS_ signal was subtracted from the aECG* signal, thus generating the fECG signal, denoted as error signal *e*(*n*).

**Fig 6:**
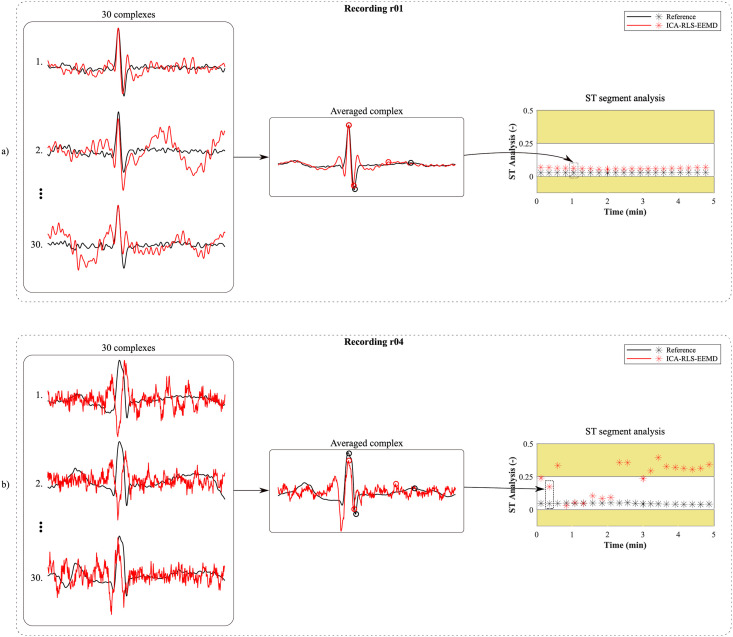
Averaging of the fQRS complexes and T/QRS calculation of a) recording r01, which achieved high accuracy; b) for recording r04, which achieved poor results.

**Fig 7:**
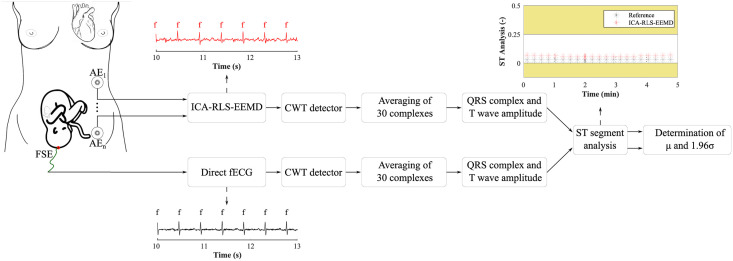
Block diagram illustrating the ST segment analysis process.

*Algorithms settings*—to obtain accurate results, it was necessary to select the most suitable combination of aECG signals and optimal hyperparameters setting of the RLS (filter order) and EEMD (*N*, *N*_*std*_, and IMFs combination) algorithms. The most suitable combination of aECG signals and parameters setting was selected on the basis of an automated algorithm. It compared all combinations of the input aECG signals and parameters setting and selected the combination that provided the highest accuracy according to the ACC measure. The comparison was carried out using the reference annotations. The best parameters values of the RLS and EEMD methods are presented in Tables 1 and 2, for each recording separately. Collumn *n* represents For the sake of clarity, the principle of the extraction system is described in the following steps:

1. First, the most appropriate combination of the aECG signals was selected for each recording. Since the ICA method is multichannel, at least two abdominal signals had to be selected. For each recording, it was investigated which combination of electrodes gave the best results based on the calculation of the ACC measure. Four abdominal signals were available for each recording; thus, 11 combinations of signals were tested. If all four signals were used for some recordings, less accurate extraction was achieved, because not all signals were recorded in high quality.
2. Subsequently, the aECG signals were filtered using a bandpass finite impulse response filter (FIR) with a range of 3–150 Hz and filter order of 500. The purpose of the filter was to remove the fluctuations of isolines.
3. The ICA method was further applied to the aECG signals. The FastICA algorithm based on kurtosis was used. The number of iterations was set to 20. The input abdominal signals (aECG) were divided into three components. In most cases, one component corresponded to the mECG signal, the second one to the aECG signal (denoted as aECG*) with the enhanced fECG component, and the third one was noise (the assignment of the components was based on the number of peaks detected). When using the ICA method, the polarity of the individual components may be inverted, see mECG in Fig 4a). This problem was solved by means of an algorithm controlling the positive and negative maxima in the interval of 50 samples from the detected R wave. In this way, the algorithm was able to detect and adjust the polarity (see Fig 4b).
4. The mECG and aECG* signals, after time and amplitude centering, were fed to the RLS system. The RLS algorithm adjusted the mECG component to the form of the parent component contained in the aECG* signal. Finally, this RLS algorithm output (denoted as mECG_RLS_) was subtracted from the aECG* signal. The output of the RLS algorithm comprised an fECG signal in which the mECG component was suppressed. The forgetting factor was set to one in order to optimize the RLS algorithm. For each recording, values from 2 to 100 with a step of two were tested to find the most appropriate filter order. Fig 5 shows the RLS filter structure with examples of the input and output signals.
5. The EEMD method, which decomposed the signal into 12 IMFs, was applied to remove the residual noise. An enhanced fECG* was obtained as the sum of the suitable IMFs. The value of standard deviation of the added noise series *N*_*std*_ was tested in the range of 0.1–0.9 with a step of 0.1, and the number of ensemble trials *N* was set to 10, 30 and 60. Testing for higher values of *N* was not necessary as there was no further improvement in the fECG extraction.
6. Finally, the fHR was determined by detecting R-peaks. The detection was done using an R-peaks detector, which was based on the continuous WT [86]. The first derivative of the Gaussian function was used as the mother wavelet. The comparison of fQRS complexes detection accuracy was done using annotations containing precisely marked positions of R-peaks. The following statistical parameters were used to evaluate the performance of the method: false positive (FP), false negative (FN) and true positive (TP). A detected fQRS complex was considered correct if its R-peak was found within 50 ms time frame before or after the reference (annotated) R-peak position [42]. Using FP, FN and TP values, it was possible to further determine the accuracy (ACC), indicating the correctness of the fQRS complexes detection, the sensitivity (SE), the positive predictive value (PPV) and F1-score indicating the overall accuracy of the filtration [27, 42]. The relevant equations are given below:
  $$\begin{array}{c} ACC=\frac{TP}{TP+FP+FN}\cdot 100\left(\%\right). \end{array}$$ (3)
  $$\begin{array}{c} SE=\frac{TP}{TP+FN}\cdot 100\left(\%\right). \end{array}$$ (4)
  $$\begin{array}{c} PPV=\frac{TP}{TP+FP}\cdot 100\left(\%\right). \end{array}$$ (5)
  $$\begin{array}{c} F1-score=\frac{2\cdot TP}{2\cdot TP+FP+FN}\cdot 100\left(\%\right). \end{array}$$ (6)
7. Bland-Altman plots were used to graphically evaluate the accuracy of fHR determination. The results of the fHR determination were compared with the values in the annotations. The 95% limits of agreement are most often used for this graph. This is an estimate of the interval *μ*±1.96*σ* in which 95% of the difference values can be expected. If the values of the differences determined lie within this interval, the difference between the methods compared is negligible [87, 88]. Also, graphical evaluation using fHR traces was done to determine the accuracy of the fHR over time. To plot the traces, it was necessary to determine the current heart rates between the individual R-peaks and to apply a moving average. The most suitable size of the moving average window was 30 samples.
8. Finally, ST segment analysis was performed on records from the FECGDARHA database. First, the signal magnitude was normalized according to the R-peak maximum, and then 30 individual cycles of fECG were averaged. This was inspired by the STAN device used in clinical practice, which also averages the T/QRS values of thirty consecutive fECG cycles. A fixed window of 600 ms (determined to cover all the physiological changes occurring) was used for averaging, namely 190 ms from the R wave to the left and 410 ms from the R wave to the right. Fig 6 shows an example of the averaging and T/QRS calculation procedure. Fig 6a shows examples of the individual fQRS complexes and their average acquired with good accuracy with respect to the reference signal (recording r01); Fig 6b shows an example of individual fQRS complexes and their average with insufficient accuracy (caused by a poor quality of the input signals) with respect to the reference (recording r04).
  To determine the T/QRS ratios, it was necessary to detect the R, S, and T waves. The beginning and end of the fQRS complex were detected using the same continuous WT detector that was used to detect R-peaks. The detector was set to four levels of decomposition and first order Gaussian mother wavelet. A different approach was used for the T wave detection. The signal was first duplicated, then a Butterworth bandpass filter was applied at frequencies of 0.5–10 Hz, (in this range, T wave has the most significant frequency representation). Subsequently, the QRS complex was zeroed and the T wave was found by thresholding. ST analysis was obtained by determining T/QRS ratios for each of the averaged fECG cycles. Fig 7 provides a block diagram illustrating the ST segment analysis process.

## Results

The results of the ICA-RLS-EEMD method are provided in this section. The evaluation of the performance was conducted in terms of the correctness of the fQRS complexes detection and the fHR determination on recordings from the FECGDARHA and Physionet Challenge 2013 database. Bland-Altman plots and fHR traces are also presented. This section also provides the results of ST segment analysis, which was carried out on the recordings from the FECGDARHA database. In the last subsection, the results of the ICA-RLS-EEMD method are compared with the results obtained in other studies.

### Detection of fetal QRS complexes

Statistical evaluation of the fQRS complexes detection accuracy was performed using annotations. First, TP, FP and FN values were determined, and based on them, the ACC, SE, PPV and F1-score indices were calculated. When testing the proposed method on the FECGDARHA database (see Table 1), the ACC higher than 95% was not noticed for recordings r04, r06, r07, r11 and r12. The SE was higher than 95% for most of the recordings except r04, r07, and r11, the PPV parameter exceeded the value of 95% for all recordings except r11, and, the F1-score was over 95% for most of the recordings except r04 and r11. For all recordings except r11, all considered quality indices exceeded the value of 80%. In the case of r01, r02 and r05 recordings, all fQRS complexes were extracted correctly and there were no false positive or negative detections, consequently all quality parameters achieved a value of 100%.

**Table 1:** Statistical evaluation of the fQRS complexes detection obtained by using the ICA-RLS-EEMD method and the FECGDARHA database (the 95% confidence interval is reported in parenthesis).

Rec.	Comb. of elect.	Filter order	N	N_std_	IMFs	n	TP	FP	FN	ACC (%)	SE (%)	PPV (%)	F1-score (%)
r01	1, 3, 4	2	10	0.5	4+7	644	644	0	0	100	100	100	100
										(99.43–100)	(99.43–100)	(99.43–100)	(99.71–100)
r02	1, 4	16	10	0.4	3+4	660	660	0	0	100	100	100	100
										(99.44–100)	(99.44–100)	(99.44–100)	(99.72–100)
r03	2, 4	86	60	0.4	2+3+4+7	684	679	1	5	99.12	99.27	99.85	99.56
										(98.10–99.68)	(98.30–99.76)	(99.18–100)	(99.05–99.84)
r04	2, 3	46	30	0.8	2+3+5	632	577	26	55	87.69	91.30	95.69	93.44
										(84.93–90.10)	(88.82–93.38)	(93.75–97.16)	(91.91–94.76)
r05	1, 4	16	10	0.5	3+4	645	645	0	0	100	100	100	100
										(99.43–100)	(99.43–100)	(99.43–100)	(99.72–100)
r06	1, 2, 3, 4	98	30	0.9	2+4+7	674	648	22	26	93.10	96.14	96.72	96.43
										(90.96–94.87)	(94.40–97.47)	(95.07–97.93)	(95.29–97.36)
r07	1, 3, 4	46	60	0.6	2+5	627	591	17	36	91.77	94.26	97.20	95.71
										(89.37–93.77)	(92.14–95.95)	(95.56–98.36)	(94.42–96.77)
r08	1, 4	30	60	0.4	4+5	651	650	0	1	99.85	99.85	100	99.92
										(99.15–100)	(99.15–100)	(99.43–100)	(99.57–100)
r09	1, 4	16	10	0.4	2+3+4	657	656	0	1	99.85	99.85	100	99.92
										(99.16–100)	(99.16–100)	(99.44–100)	(99.85–100)
r10	1, 2, 3, 4	52	30	0.9	5+8	637	630	26	7	95.02	98.90	96.04	97.45
										(93.08–96.55)	(97.75–99.56)	(94.25–97.40)	(96.43–98.24)
r11	1, 2, 3, 4	80	60	0.2	3+7	705	460	169	245	52.63	65.25	73.13	68.97
										(49.26–55.99)	(61.60–68.76)	(69.49–76.56)	(66.41–71.44)
r12	1, 2, 3, 4	100	60	0.9	2+4+8	685	659	16	26	94.01	96.20	97.63	96.91
										(91.99–95.65)	(94.49–97.51)	(96.18–98.64)	(95.85–97.77)

When testing the method on the Challenge database (see Table 2), the limit value of 95% was not achieved for the recordings a01, a02, a06, a07, a09, a10, a11, a16, a18, a20, and a21 when considering ACC, SE and F1-score, and for the recordings a02, a06, a07, a09, a11, a16, a18, and a21, when taking into account PPV. Values of ACC, SE and F1-score higher than 80% were achieved for most of the recordings except a02, a06, a07, a09, a11, a16, a18 and a21, and the PPV higher than 80% was achieved for most of the recordings except a02, a07, a09, a11, a16 and a18. For recordings a04, a05, a08, a15, a17, a22 and a25, all fQRS complexes were correctly detected, zero value of FP and FN were achieved, resulting in 100% value of all considered quality indices. For clarity and easier visualization, we provide a summary of the records from both databases were reaching threshold values (80% and 95%), see Table 3.

**Table 2:** Statistical evaluation of the fQRS complexes detection obtained by using the ICA-RLS-EEMD method and the Challenge database (the 95% confidence interval is reported in parenthesis).

Rec.	Comb. of elect.	Filter order	N	N_std_	IMFs	n	TP	FP	FN	ACC (%)	SE (%)	PPV (%)	F1-score (%)
a01	1, 2, 3	10	30	0.5	2+4+5+6+7	145	137	3	8	92.57	94.48	97.86	94.14
										(87.09–96.23)	(89.42–97.59)	(93.87–99.56)	(93.20–98.06)
a02	1, 2, 4	2	30	0.5	4+5+8	160	81	39	79	40.70	50.63	67.50	57.86
										(37.81–52.85)	(42.62–58.61)	(58.35–75.77)	(51.84–63.71)
a03	1, 4	100	10	0.1	4+6+8	128	127	1	1	98.45	99.22	99.22	99.22
										(94.51–99.81)	(95.72–99.98)	(95.72–99.98)	(97.21–99.91)
a04	1, 2	64	10	0.1	3+5+6	129	129	0	0	100	100	100	100
										(97.18–100)	(97.18–100)	(97.18–100)	(98.58–100)
a05	1,3	32	10	0.1	3+4	129	129	0	0	100	100	100	100
										(97.18–100)	(97.18–100)	(97.18–100)	(98.58–100)
a06	2, 4	24	30	0.8	2+4+8	160	106	24	54	57.61	66.25	81.54	73.10
										(50.12–64.85)	(58.36–73.52)	(73.79–87.80)	(67.61–78.12)
a07	1, 2, 3, 4	66	10	0.3	2+3+6	130	70	48	60	39.33	53.85	59.32	56.45
										(32.10–46.91)	(44.89–62.62)	(49.89–68.27)	(50.03–62.71)
a08	1, 4	100	10	0.1	3	128	128	0	0	100	100	100	100
										(97.16–100)	(97.16–100)	(97.16–100)	(98.57–100)
a09	1, 4	94	30	0.1	6+10	130	40	50	90	22.22	30.77	44.44	36.36
										(16.38–29)	(22.98–39.46)	(33.96–55.30)	(30–43.10)
a10	2, 4	98	60	0.5	3	175	149	5	26	82.78	85.14	96.75	90.58
										(76.45–87.99)	(78.99–90.06)	(92.59–98.94)	(86.89–93.51)
a11	1, 4	24	60	0.6	4+5+6+9	140	77	24	63	46.95	55	76.24	63.90
										(39.13–54.89)	(46.37–66.74)	(66.74–84.14)	(57.49–69.97)
a12	1,3, 4	14	10	0.1	2+3+4	138	136	2	2	97.14	98.55	98.55	98.55
										(92.85–99.22)	(94.86–99.82)	(94.86–99.82)	(96.33–99.60)
a13	2, 4	36	60	0.7	2+3+5	126	124	1	2	97.64	98.41	99.20	98.81
										(93.25–99.51)	(94.38–99.81)	(95.62–99.98)	(96.55–99.75)
a14	1, 2, 3, 4	10	10	0.2	4+6+9	123	120	2	3	96	97.56	98.36	97.96
										(90.91–98.69)	(93.04–99.50)	(94.20–99.80)	(95.30–99.33)
a15	1, 4	94	10	0.1	3+4	134	134	0	0	100	100	100	100
										(97.29–100)	(97.29–100)	(97.29–100)	(98.63–100)
a16	1, 4	40	10	0.7	5	130	55	54	75	29.89	42.31	50.46	46.03
										(23.38–37.07)	(33.70–51.28)	(40.72–60.18)	(39.58–52.57)
a17	1, 4	100	10	0.1	3+4	132	132	0	0	100	100	100	100
										(97.24–100)	(97.24–100)	(97.24–100)	(97.78–100)
a18	1, 2, 3, 4	34	30	0.9	3+5+6+7	150	29	80	121	12.61	19.33	26.61	22.39
										(8.61–17.60)	(13.35–26.57)	(18.60–35.93)	(17.47–27.97)
a19	3, 4	42	10	0.9	5	127	126	1	1	98.44	99.21	99.21	99.21
										(94.47–99.81)	(95.69–99.98)	(95.69–99.98)	(97.19–99.91)
a20	1, 4	96	60	0.9	5	131	117	6	14	85.40	89.31	95.12	92.13
										(78.36–90.85)	(82.72–94.03)	(89.69–98.19)	(88.10–95.12)
a21	2, 3, 4	4	60	0.7	3+6+7	145	102	11	43	65.39	70.35	90.27	79.07
										(57.36–72.81)	(62.20–77.64)	(83.25–95.04)	(73.59–83.87)
a22	1, 4	32	10	0.1	4	126	126	0	0	100	100	100	100
										(97.12–100)	(97.12–100)	(97.12–100)	(98.55–100)
a23	1, 3	36	60	0.8	5+9	126	124	1	2	97.64	98.41	99.20	98.81
										(93.25–99.51)	(94.38–99.81)	(95.62–99.98)	(96.55–99.75)
a24	1, 3	50	30	0.6	2+4+5	123	118	1	5	95.16	95.94	99.16	97.52
										(89.77–98.20)	(90.77–98.67)	(95.41–99.98)	(94.68–99.09)
a25	2, 3	94	30	0.8	2+3+5	125	125	0	0	100	100	100	100
										(97.09–100)	(97.09–100)	(97.09–100)	(98.54–100)

**Table 3:** Summary of records from both databases, which reached threshold values (80% and 95%).

Threshold values	Dataset	
	FECGDARHA	Challenge 2013
ACC > 95%	r01, r02, r03, r05, r08, r09, r10	a03, a04, a05, a08, a12, a13, a14, a15, a17, a19, a22, a23, a24, a25
ACC > 80%	r01, r02, r03, r04, r05, r06, r07, r08, r09, r10, r12	a01, a03, a04, a05, a08, a10, a12, a13, a14, a15, a17, a19, a20, a22, a23, a24 a25
SE > 95%	r01, r02, r03, r05, r06, r08, r09, r10, r12	a03, a04, a05, a08, a12, a13, a14, a15, a17, a19, a22, a23, a24, a25
SE > 80%	r01, r02, r03, r04, r05, r06, r07, r08, r09, r10, r12	a01, a03, a04, a05, a08, a10, a12, a13, a14, a15, a17, a19, a20, a22, a23, a24 a25
PPV > 95%	r01, r02, r03, r04, r05, r06, r07, r08, r09, r10, r12	a01, a03, a04, a05, a08, a10, a12, a13, a14, a15, a17, a19, a20, a22, a23, a24 a25
PPV > 80%	r01, r02, r03, r04, r05, r06, r07, r08, r09, r10, r12	a01, a03, a04, a05, a06, a08, a10, a12, a13, a14, a15, a17, a19, a20, a21, a22, a23, a24 a25
F1-score > 95%	r01, r02, r03, r05, r06, r07, r08, r09, r10, r12	a03, a04, a05, a08, a12, a13, a14, a15, a17, a19, a22, a23, a24, a25
F1-score > 80%	r01, r02, r03, r04, r05, r06, r07, r08, r09, r10, r12	a01, a03, a04, a05, a08, a10, a12, a13, a14, a15, a17, a19, a20, a22, a23, a24 a25

### Determination of fetal heart rate

Bland-Altman plots are used to show the differences between two measurements. The vertical axis of the plot shows the differences between paired signal values, while the horizontal one shows their arithmetic mean. The middle horizontal line indicates the mean *μ* of all differences. Based on this line, a 95% limits of agreement that lie in the interval *μ* ± 1.96*σ*, are plotted [87]. The results can be interpreted as follows: the smaller the range of the limits of agreement, the smaller the difference between the fHR, which is determined by the method, and the annotated signal [88]. The mean values *μ* and the values of limits of agreement determined by the ICA-RLS-EEMD method for recordings from the FECGDARHA and Challenge databases are presented in Tables 4 and 5, respectively.

**Table 4:** Mean values *μ* and values of limits of agreement determined by the ICA-RLS-EEMD method for recordings from the FECGDARHA database (the 95% confidence interval is reported in parenthesis).

Rec.	*μ* (bpm)	Upper limit of agreement (bpm)	Lower limit of agreement (bpm)
r01	-0.18	5.14	-5.50
	(-0.39 to 0.03)	(4.78 to 5.50)	(-5.85 to -5.14)
r02	-0.02	7.62	-7.66
	(-0.32 to 0.28)	(7.11 to 8.13)	(-8.16 to -7.15)
r03	-0.21	2.49	-2.91
	(-0.31 to -0.10)	(2.32 to 2.67)	(-3.01 to -2.73)
r04	-1.57	6.03	-9.17
	(-1.87 to -1.27)	(5.51 to 6.55)	(-9.69 to -8.65)
r05	0.02	3.94	-3.90
	(-0.14 to 0.18)	(3.68 to 4.21)	(-4.17 to -3.64)
r06	0.09	4.25	-4.07
	(-0.07 to 0.25)	(3.98 to 4.53)	(-4.35 to -3.80)
r07	-1.13	3.99	-6.25
	(-1.33 to -0.92)	(3.63 to 4.33)	(-6.58 to -5.89)
r08	-0.40	6.77	-7.57
	(-0.68 to -0.12)	(6.29 to 7.26)	(-8.06 to -7.09)
r09	-0.07	2.64	-2.78
	(-0.18 to 0.03)	(2.45 to 2.82)	(-2.96 to -2.60)
r10	-0.13	6.63	-6.89
	(-0.40 to 0.14)	(6.17 to 7.09)	(-7.35 to -6.43)
r11	-8.85	15.27	-32.97
	(-9.76 to -7.94)	(13.71 to 16.83)	(-34.53 to -31.42)
r12	-0.77	6.92	-8.46
	(-1.06 to -0.47)	(6.42 to 7.43)	(-8.96 to -7.95)

**Table 5:** Mean values *μ* and values of limits of agreement determined by the ICA-RLS-EEMD method for recordings from the Challenge database (the 95% confidence interval is reported in parenthesis).

Rec.	*μ* (bpm)	Upper limit of agreement (bpm)	Lower limit of agreement (bpm)
a01	-1.51	9.84	-12.86
	(-2.47 to -0.56)	(8.20 to 11.47)	(-14.49 to -11.23)
a02	-23.32	-7.88	-38.76
	(-24.55 to -22.08)	(-9.99 to -5.77)	(-40.87 to -36.64)
a03	0.35	8.00	-7.30
	(-0.33 to 1.04)	(6.83 to 9.18)	(-8.48 to -6.13)
a04	-0.06	16.45	-16.57
	(-1.54 to 1.41)	(13.93 to 18.98)	(-19.10 to -14.05)
a05	-0.01	1.62	-1.64
	(-0.15 to 0.14)	(1.37 to 1.87)	(-1.88 to -1.38)
a06	-16.35	2.54	-35.24
	(-17.86 to -14.84)	(-0.05 to 5.13)	(-37.83 to -32.66)
a07	1.62	21.60	-18.36
	(-0.16 to 3.40)	(18.55 to 24.64)	(-21.40 to -15.32)
a08	0.05	1.86	-1.76
	(-0.11 to 0.21)	(1.58 to 2.14)	(-2.04 to -1.48)
a09	-22.34	-4.30	-40.38
	(-23.94 to -20.73)	(-7.04 to -1.55)	(-43.13 to -37.63)
a10	-9.77	12.26	-31.80
	(-11.45 to -8.09)	(9.38 to 15.14)	(-34.68 to -28.92)
a11	-18.97	-3.23	-34.71
	(-20.32 to -17.61)	(-5.45 to -0.81)	(-37.13 to -32.48)
a12	0.53	5.40	-4.34
	(0.11 to 0.95)	(4.68 to 6.11)	(-5.06 to -3.62)
a13	-0.17	4.45	-4.79
	(-0.59 to 0.25)	(3.74 to 5.17)	(-5.50 to -4.07)
a14	0.16	7.06	-6.74
	(-0.47 to 0.79)	(5.98 to 8.14)	(-7.82 to -5.66)
a15	0.06	12.30	-12.18
	(-1.01 to 1.13)	(10.46 to 14.13)	(-14.01 to -10.34)
a16	-4.32	11.47	-20.11
	(-5.72 to -2.91)	(9.07 to 13.88)	(-22.51 to -17.70)
a17	0.07	5.67	-5.53
	(-0.43 to 0.56)	(4.82 to 6.52)	(-6.38 to -4.69)
a18	-35.92	-22.15	-49.69
	(-37.06 to -34.78)	(-24.10 to -20.21)	(-51.64 to -47.74)
a19	0.30	2.53	-1.93
	(0.10 to 0.50)	(2.19 to 2.87)	(-2.27 to -1.58)
a20	-4.79	9.14	-18.72
	(-6.02 to -3.55)	(7.03 to 11.26)	(-20.83 to -16.60)
a21	-14.34	1.12	-29.80
	(-15.64 to -13.04)	(-1.11 to 3.35)	(-32.02 to -27.57)
a22	0.05	2.09	-1.99
	(-0.13 to 0.24)	(1.78 to 2.41)	(-2.31 to -1.68)
a23	-0.57	2.84	-3.98
	(-0.88 to -0.26)	(2.32 to 3.37)	(-4.51 to -3.45)
a24	-1.75	2.73	-6.23
	(-2.16 to -1.34)	(2.03 to 3.43)	(-6.93 to -5.53)
a25	0.08	3.23	-3.07
	(-0.21 to 0.36)	(2.74 to 3.72)	(-3.57 to -2.59)

**Fig 8:**
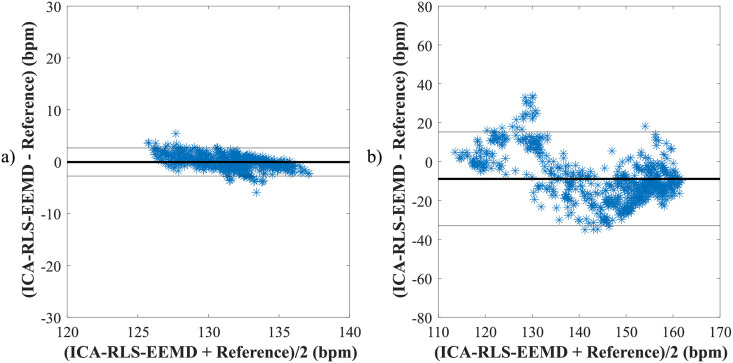
Comparison of reference and estimated values using the ICA-RLS-EEMD method when determining the fHR a) for recording r09 and b) for recording r11 based on the Bland-Altman plots.

**Fig 9:**
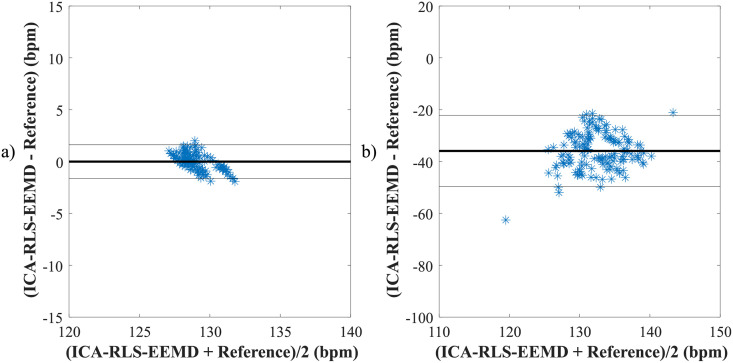
Comparison of reference and estimated values using the ICA-RLS-EEMD method when determining the fHR a) for recording a05 and b) for recording a18 based on the Bland-Altman plots.

According to Table 4, it can be noticed, that the ICA-RLS-EEMD method was effective for all recordings included in the FECGDARHA database except the recording r11, since high values of *μ* and limits of agreement were achieved. Based on the results from Table 5, it can be concluded that the method was effective for most of the recordings (except a02, a06, a09, a10, a11, a16, a18, a20, and a21) when considering values of *μ*. For the recordings a01, a04, a07 and a15, small values of *μ* were achieved as well, however, accompanied with high values of limits of agreement. Fig 8 shows Bland-Altman plots for recordings from the FECGDARHA database. Plot a) presents a recording characterized by high quality of fHR determination, while plot b) shows an example of the recording for which the ICA-RLS-EEMD method was not effective. Fig 9 illustrates Bland-Altman plots for recordings from the Challenge database. As in the previous case, plot a) presents a recording of high quality of fHR determination, whereas plot b) is an example of recording in which the ICA-RLS-EEMD method was not effective.

In order to plot fetal heart rate traces, it is necessary to determine the fHR value between the consecutive R-peaks. Based on the moving averages, fHR traces were created and compared with annotations of R-peaks determined by experts. The graphical representation used is inspired by FIGO classification [89]. The interval of fHR physiological values (110–150 bpm) is marked in white, the intervals of fHR with an increased risk of hypoxia (90–110 bpm and 150–180 bpm) are marked in yellow and the area of fHR with a high risk of hypoxia (below 80 bpm and above 180 bpm) is marked in pink [46].

**Fig 10:**
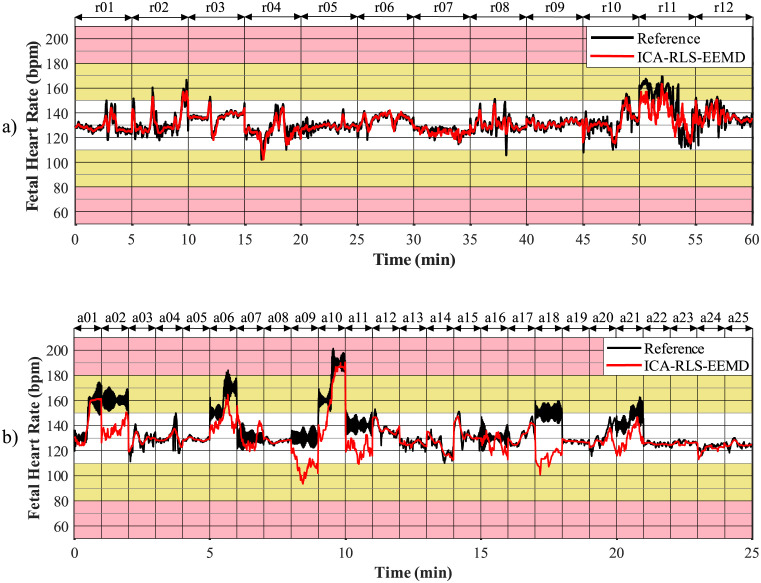
Comparison of fHR traces extracted using the ICA-RLS-EEMD method with annotation a) for all recordings from the FECGDARHA database and b) for 25 recordings from the Challenge database.

A comparison of fHR traces for both databases is shown in Fig 10. The top arrows indicate the names of the recordings related to the given section of the graph. With the FECGDARHA database, all 12 recordings having a length of five minutes were displayed, i.e. 60 minutes of recordings are shown in total. With the Challenge database, 25 recordings having a length of one minute were analysed, i.e. 25 minutes of the analysis are shown in total. Fig 10a) shows the fHR traces determined using the signals from the FECGDARHA database. It can be stated that all of the signals except the recording r11 copy the trend of the reference trace, and thus the determination of fHR was accurate. On the other hand, for the recording r11, the deviation of the resulting fHR trace from the reference one can be noticed, which means that the determination of fHR was not accurate. Moreover, based on the analysis of Fig 10b) we can state that the traces representing recordings a02, a06, a07, a09, a10, a11, a16, a18, and a21 from the Challenge database do not copy the trend of the reference trace and the method was thus not effective in determining the fHR for these recordings. For the recordings a01 and a20, a slight deviation of the resulting curve from the reference one can be noticed, which means that the fHR was not determined accurately using this method in some parts of these recordings.

### ST segment analysis

For evaluation of the ST segment analysis accuracy, we compared the T/QRS ratios estimated using the method with those determined using the reference signals acquired by means of fetal scalp electrode. For the comparison, we used the mean values *μ* and values of limits of agreement which are summarized in Table 6. Low mean values *μ* and values of limits of agreement were obtained for recordings r01, r02, r03, r05, r08, r09, and r10, showing a high accuracy of ST segment analysis. Contrary, in recordings r04, r06, r07, r11, and r12, the mean values *μ* and values of limits of agreement reached high values and thus the ST segment analysis was inaccurate.

**Table 6:** Mean values *μ* and values of limits of agreement determined for ST segment analysis (the 95% confidence interval is reported in parenthesis).

Rec.	*μ* (-)	Upper limit of agreement (-)	Lower limit of agreement (-)
r01	0.0320	0.0408	0.0232
	(0.0299 to 0.0340)	(0.0372 to 0.0443)	(0.0196 to 0.0267)
r02	0.0389	0.0615	0.0163
	(0.0338 to 0.0440)	(0.0526 to 0.0703)	(0.0074 to 0.0251)
r03	0.0121	0.0241	0.0001
	(0.0094 to 0.0149)	(0.0194 to 0.0288)	(-0.0045 to 0.0049)
r04	0.2027	0.4688	-0.0634
	(0.1409 to 0.2645)	(0.3614 to 0.5761)	(-0.1707 to 0.0439)
r05	0.0277	0.0588	-0.0034
	(0.0205 to 0.0350)	(0.0463 to 0.0713)	(-0.0159 to 0.0092)
r06	1.2173	2.4592	-0.0246
	(0.9364 to 1.4983)	(1.9716 to 2.9468)	(-0.5122 to 0.4630)
r07	1.5565	3.4169	-0.3039
	(1.1122 to 2.0007)	(2.6446 to 4.1890)	(-1.0761 to 0.4683)
r08	-0.0126	0.0051	-0.0303
	(-0.0167 to -0.0085)	(-0.0020 to 0.0122)	(-0.0374 to -0.0231)
r09	0.0167	0.0285	0.0049
	(0.0139 to 0.0194)	(0.0237 to 0.0332)	(0.0001 to 0.0096)
r10	0.0137	0.0262	0.0012
	(0.0108 to 0.0166)	(0.0212 to 0.0312)	(-0.0039 to 0.0062)
r11	5.7123	14.0104	-2.5858
	(3.8815 to 7.5431)	(10.8350 to 17.1857)	(-5.7611 to 0.5895)
r12	2.3200	4.0628	0.5772
	(1.9258 to 2.7143)	(3.3786 to 4.7470)	(-0.1070 to 1.2615)

**Fig 11:**
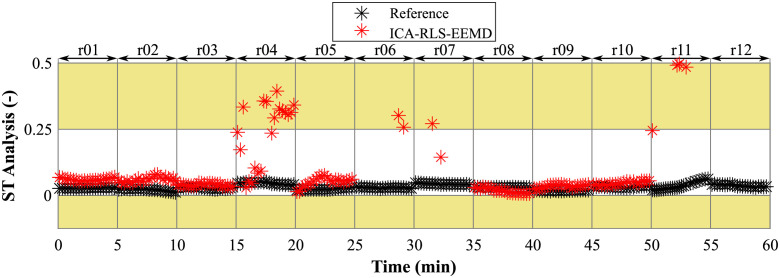
Comparison of estimated and reference T/QRS ratios of averaged fECG complexes over time.

These results were confirmed by plotting the estimated and reference T/QRS ratios of the averaged fECG complexes over time, see Fig 11. Accurate ST segment analysis was achieved in 7 of the 12 records tested (r01, r02, r03, r05, r08, r09 and r10). For the remaining five records (r04, r06, r07, r11, and r12), the ST segment analysis was not effective—the estimated values significantly differ from the reference ones. For records r06, r07, r11, and r12, the deviations of the estimated T/QRS ratios from the reference T/QRS ratios were so large that most of the red data points in these records were outside the y-axis range and are therefore not visible.

### Comparison of the results

In this part, the results provided using the ICA-RLS-EEMD method are compared with the results of other methods reported in other studies. Such a comparison is very difficult, as a large number of various databases and different recordings are used for testing. They include, for example, the *Database for the Identification of Systems* (DaISy) [90], used in [29, 33, 39, 40], the *Fetal ECG Synthetic Database* (FECGSYNDB) [50], used in [91, 92], or the *Non-Invasive Fetal ECG Database* (NIFECGDB) available on the *PhysioNet* website [84], applied in [36, 44, 67]. There are also studies [26, 37], in which the authors do not specify on which data they tested their methods, and, in some studies, i.e. [38], authors use their own set of signals. In terms of evaluating the effectiveness of the method, various quality indices are applied. In [38] the SNR was used, in [93] the MSE was applied, while in [94] authors used the correlation coefficient. In addition, a number of studies [28, 29, 35, 67, 95], do not use any statistical evaluation of the effectiveness, presenting examples of extracted signals only. Other studies, like [31], test their methods only on a limited number of signal samples. For these reasons, we made both an objective and a subjective comparison of the results summarized in Table 7.

- A database containing the same recordings as the FECGDARHA is called *Abdominal and Direct Fetal ECG Database* (ADFECGDB) and is available in *Physionet* [84], but contains only five recordings (r01, r04, r07, r08 and r10). In [96], the authors tested a combination of compressive sensing (CS) and the ICA method. The method was tested only on these five recordings from the ADFECGDB database and the SE, PPV and F1-score were used to evaluate the extraction quality. The study achieved 92.20% average accuracy of R-peaks detection according to F1-score parameter. This is a worse result compared to the ICA-RLS-EEMD when tested on the FECGDARHA database, but better when tested on the Challenge database.
- In [97], the authors applied a combination of WT and a clustering-based technique (CT). The effectiveness of the method was also tested only on the recordings from the ADFECGDB database (whereas we used seven additional signals published later in [83]), and on 26 recordings from the Challenge 2013 database, where 12 of those records were the same as in our study. The effectiveness of the method was evaluated using ACC, SE, PPV, and F1-score parameters. The authors of the study achieved an average accuracy of 98.63% in the R-peak detection according to F1-score when tested on the ADFECGDB database, and 94.77% when tested on the Challenge database, which is a better result in both cases than we achieved. In general, however, the WT-CT method was less effective for recordings with higher noise levels.
- In [98], a combination of de-shape short time Fourier transform (STFT) and nonlocal median (NM) was investigated. The method was tested on recordings r01, r04, r07, r08 and r10 from the ADFECGDB database and on 75 recordings from Challenge 2013 database (including 25 records that we used herein). The authors achieved and average accuracy of 98.86% in R-peaks detection according to F1-score when tested on the ADFECGDB database; when tested on the Challenge database, they achieved an average accuracy of 86.31%, both slightly outperforming our method. However, the authors state that the P and T waves “were buried in the noise” when using the STFT-NM and thus their extraction would be nearly impossible.
- In [32], the authors tested the combination of ICA-EEMD-WS on five real recordings from the ADFECGDB database and on 500 synthetic recordings generated using the *Fetal ECG Synthetic (FECGSYN) generator* introduced in [101]. Unfortunately, the authors used other parameters (SNR, MSE) to evaluate the accuracy of the extraction. However, according to the visual evaluation, it can be stated that the ICA-EEMD-WS was as effective as the method we proposed. According to the authors, the limitations of the ICA-EEMD-WS method include low computational speed and significant noise suppression affecting the morphology of the output signal, which can affect the clinical information contained in it.
- In [99], the authors introduced a combination of CS and non-negative matrix factorization (NMF). The method was tested on five recordings from the ADFECGDB database and 60 recordings from the Challenge 2013 database (including 25 records that we used for testing). The effectiveness of the method was evaluated using the parameters SE, PPV, and F1-score. The authors achieved an average accuracy of 94.80% in R-peaks detection according to F1-score when tested on the ADFECGDB database; when tested on the Challenge database, they achieved an average accuracy of 84%, which is the same result we achieved.
- The combination of EKS, ANFIS and differential evolution (DE) was tested in [100]. The authors tested the method on 75 records from the Challenge 2013 database and 55 records from the NIFECGDB database. The effectiveness of the method was evaluated according to the parameters ACC, SE, PPV, and F1-score. When testing on the Challenge database, the authors achieved an average accuracy of 91.82%, which is a better result than ours, and when testing on the NIFECGDB database, the average accuracy was 95.12%. According to the authors, the limitation of the method is that it may perform worse in the extraction of the fECG at lower sampling frequency compared to the extraction of the fECG at higher sampling frequency.
- Authors of the study introduced in [42] tested the combination of ICA a QIO methods. They used records from the Challenge database and from the FECGSYNDB database for testing. They used the parameters SE, PPV, and F1-score to evaluate the quality of extraction. The authors achieved an average accuracy of 99.38% according to the F1-score when testing on the Challenge database and an average accuracy of 98.78% when testing on the FECGSYNDB database, thus outperforming our method. However, the authors state that this is a low computational speed algorithm that cannot extract fECG signals in multiple pregnancy.
- In [45] the authors tested the combination of ICA and self-correlation analysis (SCA) methods on the recordings from the ADFECGDB database. The authors did not use any statistical parameters for the evaluation, and in addition, they tested the method on only five signals. However, according to the visual comparison of the extracted signals, it can be stated that our method was able to suppress the mECG component better.
- The combination of the EMD and correlation analysis (CA) was introduced in [31] and tested on signals from the ADFECGDB database. The authors evaluated the effectiveness of the method using the ACC, SE, and PPV parameters. However, since they reached 100% for all parameters, it can be assumed that the F1-score would also acquire an accuracy of 100%. In this case, however, these are very inconclusive results, as the method was tested only on four signals with a length of ten seconds.

**Table 7:** Comparison of the results with other studies.

Author, source	Algorithm	Dataset	Average accuracy of R-peaks detection according to F1-score (%)	Advantages and limitations
Da Poian et al. [96]	CS-ICA	ADFECGDB	92.20	+ the algorithm works in real time
				- tested on a small number of records
Castillo et al. [97]	WT-CT	ADFECGDB Challenge 2013	98.63	+ allows use in automated applications in real time
			94.77	- less effective for recordings with higher noise levels
Su et al. [98]	STFT-NM	ADFECGDB Challenge 2013	98.86	+single channel method
			86.31	- P wave and T wave could not be extracted
Liu et al. [32]	ICA-EEMD-WS	ADFECGDB FECGSYN	–	+ automated selection of suitable IMFs
				- suppression of clinical information in the signal
				- low computational speed
Gurve et al. [99]	CS-NMF	ADFECGDB Challenge 2013	94.80	+ single channel method
			84	+ the use of CS could lead to a low-power monitoring system
				- less effective for recordings with higher noise levels
Panigrahy et al. [100]	EKS-DE-ANFIS	Challenge 2013 NIFECGDB	91.82	+ single channel method
			95.12	+ does not require parameters initialization
				- lower performance for at lower sampling frequency
Billeci et al. [42]	ICA-QIO	Challenge 2013 FECGSYNDB	99.38	+ effective even for very noisy signals
			98.78	- low computational speed
				- unsuitable for a twin pregnancy
Li et al. [45]	ICA-SCA	ADFECGDB	–	+ high computational speed
				- tested on a small number of records
Azbari et al. [31]	EMD-CA	ADFECGDB	100	+ effective on signals of different quality
				- tested on a small number of records
				- tested on very limited signal lengths
Proposed algorithm	ICA-RLS-EEMD	FECGDARHA Challenge 2013	95.69	+ allows deeper morphological analysis
			84.08	- computational complexity

We also compared the results obtained using the conventional ICA method alone, for all recordings from both the FECGDARHA and Challenge databases were better results achieved using the proposed procedure. Using the ICA-RLS-EEMD method and the FECGDARHA database, ACC >80% was achieved for 11 out of 12 recordings with an average value of ACC = 92.75% [95% confidence interval: 91.19–93.88%], SE = 95.09% [95% confidence interval: 93.68–96.03%], PPV = 96.36% [95% confidence interval: 95.05–97.17%] and F1-score = 95.69% [95% confidence interval: 94.83–96.35%], while, using the conventional ICA method, ACC >80% was achieved for 3 out of 12 recordings with an average value of ACC = 47.77% [95% confidence interval: 45.38–50.12%], SE = 57.68% [95% confidence interval: 55.04–60.23%], PPV = 57.59% [95% confidence interval: 54.39–60.76%] and F1-score = 57.37% [95% confidence interval: 55.92–59.38%]. Using the proposed method and the Physionet Challenge 2013 database, ACC >80% was achieved for 17 out of 25 recordings with an average value of ACC = 78.24% [95% confidence interval: 73.44–81.85%], SE = 81.79% [95% confidence interval: 76.59–85.43%], PPV = 87.16% [95% confidence interval: 81.95–90.35%] and F1-score = 84.08% [95% confidence interval: 80.75–86.64%], while, using the conventional ICA method, ACC >80% was achieved for 6 out of 25 recordings with an average value of ACC = 38.72% [95% confidence interval: 31.88–41.25%], SE = 48.26% [95% confidence interval: 40.37–52.66%], PPV = 49.35% [95% confidence interval: 42.41–53.78%] and F1-score = 48.32% [95% confidence interval: 41.94–50.51%].

Finally, we performed a further comparison using methods which were presented in our previous studies. In [46], we introduced the ICA-ANFIS-WT and ICA-RLS-WT methods and, in [47], we tested the ICA-EMD, ICA-EMD-WT and ICA-RLS-EMD procedures. We applied the same testing routine as presented in this work, i.e. using the FECGDARHA and Challenge databases, and the same set of quality indices i.e., ACC, SE, PPV and F1-score. Using the ICA-RLS-EEMD method, we achieved higher values of considered indices for most of the recordings. The exceptions are the recording a07 from the Challenge database, where better results were provided for the ICA-ANFIS-WT method, and the recording a21, for which the ICA-ANFIS-WT and ICA-RLS-WT methods provided better results. The average values of the ACC, SE, PPV and F1-score parameters, which were obtained using all our previous methods along with results of the conventional ICA, are summarized in Table 8 (the FECGDARHA database) and Table 9 (the Challenge database). The second column of the tables shows the number of recordings for which the ACC parameter value was higher than 80%.

**Table 8:** Comparison of performance of the methods when extracting the fECG signals from the FECGDARHA database. Second column provides the number of recordings for which ACC >80% was achieved. Columns three to six show average values calculated for all 12 recordings from the database (the 95% confidence interval is reported in parenthesis).

Methods	ACC >80%	ACC (%)	SE (%)	PPV (%)	F1-score (%)
ICA	3	47.77	57.68	57.59	57.37
		(45.38–50.12)	(55.04–60.23)	(54.39–60.76)	(55.92–59.38)
ICA-EMD	5	48.22	52.66	60.02	55.71
		(46.11–50.28)	(50.12–55.16)	(57.01–62.95)	(53.80–57.60)
ICA-EMD-WT	5	51.91	58.05	64.08	61.03
		(49.51–54.25)	(55.27–60.75)	(61.81–67.64)	(59.02–62.99)
ICA-ANFIS-WT	6	64.34	71.05	76.30	73.29
		(62.06–65.04)	(68.51–73.38)	(73.58–78.75)	(71.48–74.98)
ICA-RLS-EMD	9	84.73	87.99	92.72	90.10
		(82.76–86.34)	(86.08–89.52)	(90.96–94.01)	(88.85–91.16)
ICA-RLS-WT	9	85.92	89.70	92.41	90.99
		(83.85–87.64)	(87.76–91.24)	(90.69–93.68)	(89.73–92.04)
**ICA-RLS-EEMD**	**11**	**92.75**	**95.09**	**96.36**	**95.69**
		**(91.19–93.88)**	**(93.68–96.03)**	**(95.05–97.17)**	**(94.83–96.35)**

**Table 9:** Comparison of performance of the methods when extracting the fECG signals from the Challenge database. Second column provides the number of recordings for which ACC >80% was achieved. Columns three to six show average values calculated for 25 recordings from the database (the 95% confidence interval is reported in parenthesis).

Methods	ACC >80%	ACC (%)	SE (%)	PPV (%)	F1-score (%)
ICA	6	38.72	48.26	49.35	48.32
		(31.88–41.25)	(40.37–52.66)	(42.41–53.78)	(41.94–50.51)
ICA-ANFIS-WT	7	59.51	67.09	72.76	69.44
		(51.41–62.81)	(58.68–71.30)	(64.53–77.53)	(63.27–72.25)
ICA-RLS-EMD	12	64.95	69.34	79.62	72.74
		(60.29–70.99)	(64.44–75.70)	(74.52–85.82)	(70.58–78.50)
ICA-RLS-WT	13	68.25	72.60	81.31	75.68
		(62.70–72.76)	(66.87–77.12)	(74.77–86.15)	(71.65–79.06)
**ICA-RLS-EEMD**	**17**	**78.24**	**81.79**	**87.16**	**84.08**
		**(73.44–81.85)**	**(76.59–85.43)**	**(81.95–90.35)**	**(80.75–86.64)**

## Discussion

The results presented in the previous section demonstrate the ICA-RLS-EEMD method capability to extract high-quality fECG signals. For most of the recordings tested, the method eliminated the maternal component very effectively and determined the fHR with high accuracy. The maternal component was eliminated mainly with the help of the RLS method, and the subsequent application of the EEMD further improved the resulting filtration. Unfortunately, for some signals we were not able to sufficiently suppress the maternal component. In this section we discuss the reasons behind the differences in the quality of the extraction outcomes.

**Fig 12:**
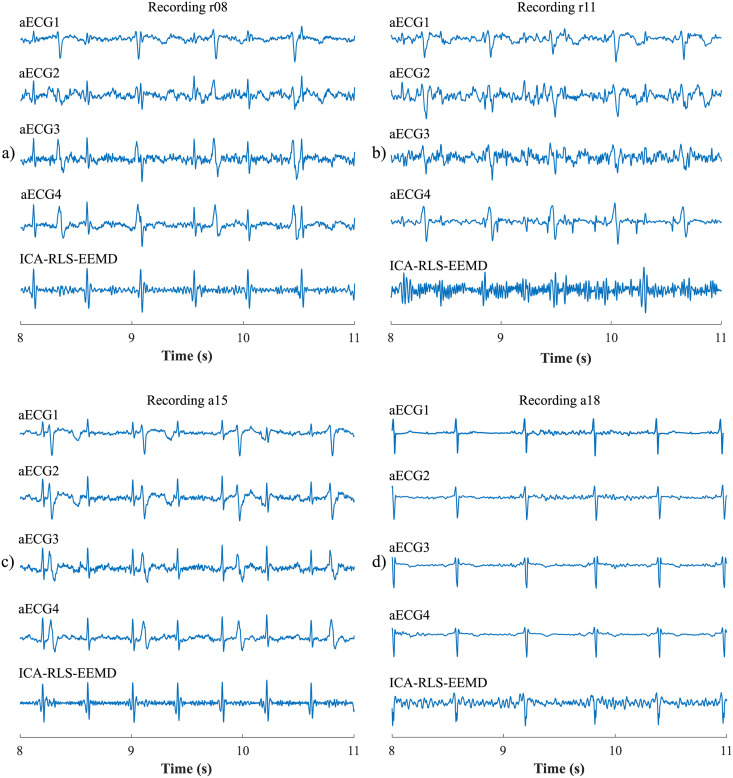
Examples of input aECG signals with different quality and the corresponding outcomes of the ICA-RLS-EEMD method. Subfigures a) and b) show the examples of the high- (r08) and low-quality (r11) recordings from the FECGDARHA database, for which, respectively, high and low accuracy of the ICA-RLS-EEMD extraction was noticed. Subfigures c) and d) show examples of high- (a15) and low-quality (a18) recordings from the Challenge database, from which fECG was and was not successfully extracted using the ICA-RLS-EEMD, respectively.

Studies [1, 102, 103] demonstrated a significant impact of electrode placement and data acquisition quality on the final fECG extraction. An example of such influence is shown in Fig 12. Example a) shows the recording r08 from the FECGDARHA database and example c) is an illustration of recording a15 from the Challenge database. In both of them, it can be seen that the magnitude of the fetal component is sufficient compared to the maternal one. In these cases, extraction by the system was highly effective, and, in both cases, ACC >99% was achieved when detecting the fQRS complexes. Example b) illustrates the recording r11 from the FECGDARHA database being an example of aECG signals of lower quality. In this case, the magnitude of the fetal component is very low compared to the maternal component and, in addition, the signal contains noise. From such input signals, it is difficult to extract a good quality fECG signal and to accurately detect the fQRS complexes. As a result, the reported ACC was less than 53%. Finally, example d) of recording a18 from the Challenge database is presented. Here, the ICA-RLS-EEMD method completely failed to extract the fECG as the ACC of the QRS detection and the determination of the fHR was below 13%. In such aECG signals the magnitude of the fetal component is almost negligible and hence, the fECG extraction is difficult or practically impossible. Such input aECG signals are not usable for the methods of NI-fECG extraction.

**Fig 13:**
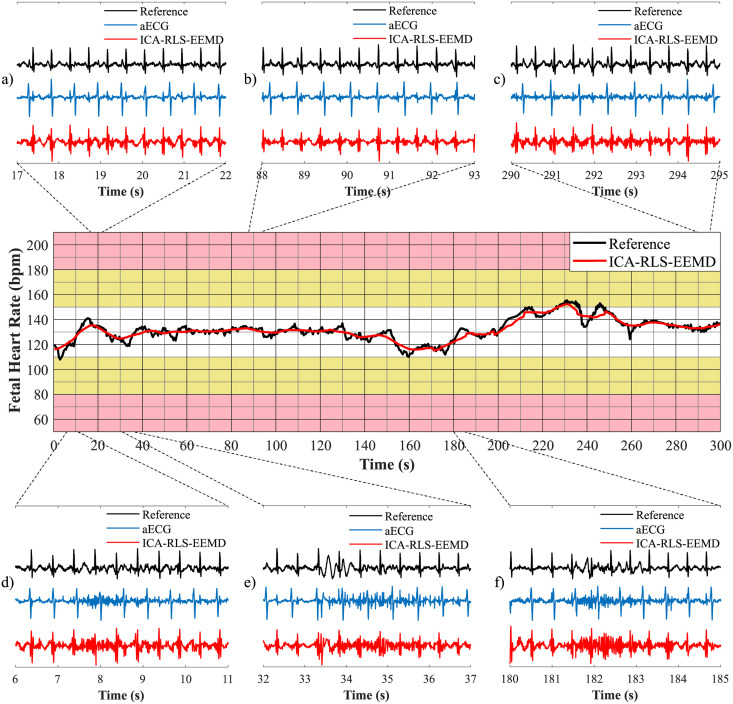
Illustration of the influence of the quality of aECG recordings on the final extraction and the fHR determination. As an example, the recording r10 from the FECGDARHA database was selected, which was characterized with the overall high-quality extraction. Traces obtained using the ICA-RLS-EEMD method are compared with the annotation. Examples a), b) and c) correspond to sections where high accuracy was achieved in the fHR determination, and examples d), e) and f) correspond to sections where the fHR determination was less accurate.

Fig 13 shows the influence of the quality of aECG on the extracted signal by comparing the fHR traces determined using the ICA-RLS-EEMD method with the reference fHR signal derived from the scalp electrode. Examples of abdominal, extracted and reference ECG signals from the scalp electrode are also presented. To best illustrate this effect, the recording r10, characterized by an overall high-quality extraction (ACC >95%), was selected from the FECGDARHA database. Examples a), b) and c) correspond to the signal sections where the fHR determination achieved high accuracy. These parts of aECG signals contain well recorded fetal component. Examples d), e) and f) correspond to sections where the estimated fHR trace deviates slightly from the annotation curve and where, as a result, the fHR determination was less accurate. This could be due to the noise which caused a slight decrease in the quality of the extraction. However, these are insignificant deviations that do not affect the final accuracy of fetal hypoxia determination.

**Fig 14:**
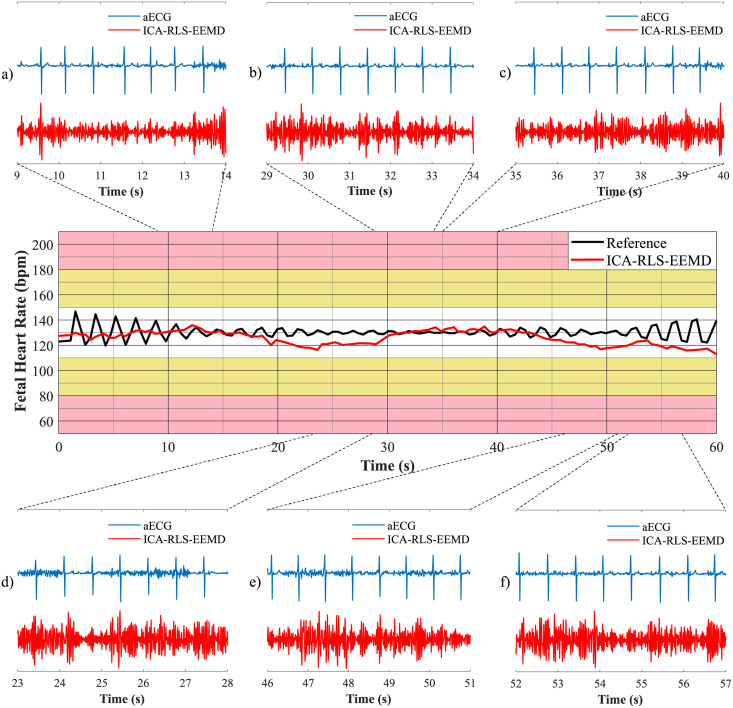
Illustration of the influence of the quality of aECG recordings on the final extraction and the fHR determination. As an example, the recording a16 from the Challenge database was selected, which was characterized by an overall low-quality extraction. Examples a), b) and c) correspond to signal sections where very low accuracy was achieved in the fHR determination, and examples d), e) and f) correspond to sections where the method failed completely when extracting the fHR.

Fig 14 shows another example of the impact of the quality of the aECG signals on the resulting extraction quality and the subsequent fHR determination. The recording a16, for which unsatisfactory results were obtained (ACC <30%), was selected from the Challenge database. In this case, only the aECG signal and the signal extracted using the ICA-RLS-EEMD method are presented, as the scalp electrode reference recording is not available. Only the annotation used to plot the fHR trace is included. Subfigures a), b) and c) correspond to sections where the method curve deviates the least from the annotation curve, but, even in these sections, sufficient extraction quality was not achieved. This is due to the very low magnitude of the fetal component. In examples d), e) and f), the complete failure of the proposed extraction method can be seen, as the fetal component is almost invisible in the aECG signal. Again, it is clear that such signals are not suitable for fECG extraction and their filtration is not feasible.

As shown, the quality of aECG signals is one of the most important factors affecting the resulting filtration quality and the accuracy of fHR determination. It can be stated that the extraction is effective if the ratio between the fetal and maternal component in the aECG record is sufficiently high and the amount of noise in the recording is low. One of the factors in aECG signal quality is the electrode placement. It affects the signal’s polarity, morphology, ratio between the maternal and fetal component or even the ability of the system to suppress the unwanted signals (e.g. movement and muscle artifacts). Unfortunately, there is currently no standardization for non-invasive fECG measurement in terms of the number, location of electrodes, or system configuration, as is the case of conventional ECG. The recordings in the publicly available databases (including the FECGDARHA and Challenge 2013) differ in the number and location of the electrodes on the maternal body. As a result, the quality of the signals may differ significantly. This effect can be observed especially in the case of the results achieved using the data from the Challenge 2013 database, which differ significantly from case to case (e.g. for the record a04 ACC = 100% whereas for a18, ACC = 12.61%). These significant differences may be caused by the fact that this database is known to be very inconsistent; it includes records of different quality, from different sources and acquired using a variety of devices with different resolutions, configurations and electrode placements. Large amount of the records are also synthetic. In addition, almost no information on the setting of the measuring system, gestational age or the position of the fetus for individual records is publicly available.

The future research should therefore aim to find the optimal number and location of electrodes, which would be standardized with respect to the position and gestational age of the fetus. It can be assumed that the more electrodes used for sensing, the more likely it is that high quality aECG signals will be captured. However, this assumption is only theoretical, as from a practical point of view, recording many signals simultaneously is both clinically and technically demanding. In addition, for the signals to be of high quality, the patient’s skin needs to be properly cleaned and the sensor attached well, which is not only time consuming in the case when many electrodes are used, but also stressful and uncomfortable for the pregnant woman. The electrodes can be placed on the abdominal area (for aECG measurement) or also on the chest (to obtain mECG). Purely abdominal sensing is beneficial for the clinical staff due to decreased requirements for preparation but also for the mother since it offers greater comfort and mobility. On the other hand, the combination of abdominal and thoracic electrodes allows easier interpretation of mECG and fECG signals. However, the effectiveness of the combined electrode placement is strongly dependent on the quality of the chest signals, which is often insufficient in clinical practice. In addition, change in the location of the sensor leads to variations in the morphology of the mECG signal which can then differ from the maternal component obtained in the abdominal area. Thus, the extraction may not be a sufficiently accurate [1, 5]. This is especially true for adaptive algorithms such as RLS, which are very susceptible to the quality of input signals, especially mECG.

**Fig 15:**
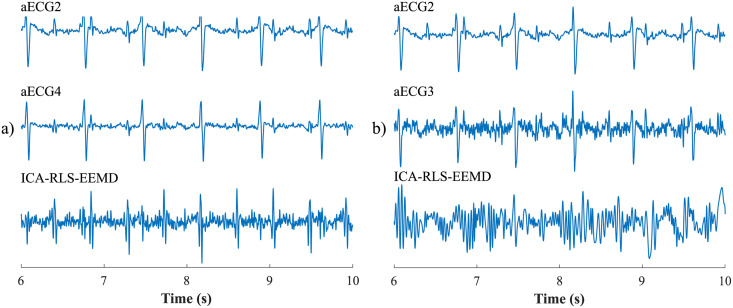
An example of the effect of the input aECG signal selection on the resulting signal quality: a) optimal selection of input aECG signals (aECG2 and aECG4), the resulting extracted signal is of a high quality; b) inappropriate combination of input signals (aECG2 and aECG3) causing the method to fail to extract fECG signals.

The influence of the performance of the ICA, RLS, EEMD individual algorithms and selection of a combination of aECG signals on the resulting quality of the outcome signal is also presented. Fig 15 shows an example of the effect of the input aECG signal selection on the resulting signal quality. Example a) presents the optimal selection of input aECG signals (aECG2 and aECG4), when the resulting extracted signal was of very high quality and ACC > 99% was achieved, while b) shows an example of inappropriate combination of input signals (aECG2 and aECG3) causing the method to fail to extract fECG signals (ACC < 30%).

In addition to the *subjective* evaluation of the input aECG waveforms and their influence on the resulting extraction quality, we also performed an *objective* statistical evaluation, see the summary in Table 10. We have introduced four objective parameters and evaluated their influence on the accuracy of fQRS complex detection:

1. *Number of input channels*—this factor was related to the theoretical assumption that the quality of the resulting extraction does not depend on the number of channels used, but on their quality and suitability in terms of ECG curve morphology. The resulting correlation coefficient was -0.28 [95% confidence interval: -0.55 to 0.05], indicating a very low negative correlation between the number of input aECG signals and the resulting accuracy in detecting fQRS complexes. For this reason, it was also necessary to qualitatively evaluate the input aECG channels using objective evaluation parameters.
2. *mR:fR ratio*—this parameter assessed whether the fetal component in a given abdominal lead reaches a sufficient level compared to the maternal component. For each record, we calculated the average ratio of the voltage levels of the detected *mR* and *fR* peaks.
  - First, all *mR* and *fR* peaks were detected in each aECG signal for each recording and their voltage levels were determined.
  - The average amplitude of the *mR* and *fR* oscillations was determined and the average *mR:fR ratio* was calculated for each aECG signal.
  - The resulting ratio was determined as the average of the ratios of all aECG signals used.
  The value of the correlation coefficient was -0.79 [95% confidence interval: -0.89 to -0.62], indicating a high negative correlation between the average *mR:fR ratio* and the resulting accuracy in detecting fQRS complexes according to ACC (i.e. the smaller the ratio between *mR* and *fR* peaks, the higher the ACC value).
3. *Skewness signal quality index (sSQI)*—this parameter is often used in the ECG signal quality evaluation in adults, see [104, 105]. The resulting value for a given record was determined as the average value of all aECG signals used. In the case of *sSQI*, a slight positive correlation was observed with the ACC values with a correlation coefficient value of 0.59 [95% confidence interval: 0.32 to 0.76].
4. *Kurtosis signal quality index (kSQI)*—similarly to *sSQI*, this parameter is used to assess the quality of ECG signal in adults. Both parameters were designed as indices to identify outliers in the ECG that are equivalent to noise, see [104, 105]. In the case of *kSQI*, a high negative correlation was observed with ACC values with a correlation coefficient value of -0.82 [95% confidence interval: -0.90 to -0.67].

**Table 10:** Analysis of the influence of four factors (number of input aECG signals, average ratio between *mR* and *fR* oscillations, average value of *sSQI* and *kSQI*) on the resulting extraction quality. The influence of each factor on the accuracy was evaluated using a correlation coefficient (the 95% confidence interval is reported in parenthesis).

Recordings	Number of input aECG signals (-)	mR:fR ratio (-)	sSQI (-)	kSQI (-)
r01	3	1.90 (1.40 to 2.86)	-0.15 (-2.66 to 1.37)	12.71 (10.00 to 17.35)
r02	2	2.05 (1.37 to2.73)	-0.60 (-2.42 to 1.23)	13.31 (10.35 to 16.28)
r03	2	2.63 (2.29 to2.96)	-1.37 (-1.63 to -1.11)	14.16 (12.87 to 15.46)
r04	2	2.42 (2.35 to 2.48)	0.49 (-0.36 to 1.33)	12.82 (12.22 to 13.42)
r05	2	1.94 (1.21 to2.67)	-0.64 (-2.35 to 1.07)	12.84 (9.78 to 15.90)
r06	4	2.46 (1.98 to 2.83)	-1.33 (-2.13 to -0.30)	16.60 (11.88 to 25.75)
r07	3	2.66 (2.25 to 3.12)	1.23 (-0.95 to 3.37)	14.57 (10.75 to 20.35)
r08	2	1.83 (1.07 to2.59)	-0.60 (-2.24 to 1.03)	12.55 (9.58 to 15.52)
r09	2	1.89 (1.09 to 2.68)	-0.61 (-1.93 to 0.72)	10.73 (8.29 to 13.18)
r10	4	2.41 (2.03 to 3.05)	-1.37 (-2.09 to -0.80)	11.99 (8.79 to 13.91)
r11	4	2.15 (1.91 to 2.45)	-0.73 (-1.13 to -0.39)	15.15 (7.53 to 21.74)
r12	4	2.90 (2.16 to 3.49)	-1.42 (-2.26 to -0.40)	19.16 (17.96 to 27.28)
a01	3	5.96 (3.84 to 8.85)	-1.71 (-2.81 to -0.45)	15.72 (13.73 to 17.49)
a02	3	5.33 (4.18 to 6.36)	-2.32 (-3.36 to -0.33)	21.70 (19.49 to 23.25)
a03	2	2.52 (2.19 to 2.85)	-1.47 (-2.00 to -0.94)	13.03 (12.57 to 13.49)
a04	2	1.83 (0.72 to 2.94)	-0.99 (-2.84 to 0.85)	12.64 (8.82 to 18.46)
a05	2	1.99 (1.24 to 2.73)	-0.85 (-2.58 to 0.88)	12.68 (8.93 to 16.42)
a06	2	5.87 (5.38 to 6.35)	-3.11 (-3.15 to -3.07)	18.35 (17.91 to 18.80)
a07	4	4.74 (3.43 to 7.05)	-1.60 (-2.63 to -0.57)	13.49 (11.88 to 15.78)
a08	2	1.85 (0.93 to 2.76)	-0.71 (-2.45 to 1.02)	13.10 (9.32 to 16.87)
a09	2	4.89 (4.09 to 5.69)	-2.42 (-4.06 to -0.78)	26.11 (22.88 to 29.34)
a10	2	4.79 (4.20 to 5.37)	-3.15 (-3.20 to -3.10)	18.20 (17.78 to 18.62)
a11	2	4.64 (4.17 to 5.10)	-2.34 (-4.01 to -0.67)	25.56 (22.38 to 28.74)
a12	3	2.76 (2.25 to 3.45)	-1.55 (-2.24 to -1.08)	13.02 (9.28 to 15.10)
a13	2	3.39 (2.87 to 3.91)	1.46 (-0.48 to 3.40)	17.56 (14.62 to 20.51)
a14	4	2.24 (1.85 to 2.84)	-1.20 (-1.99 to -0.60)	11.06 (8.49 to 12.73)
a15	2	1.79 (0.97 to 2.62)	-0.56 (-2.19 to 1.06)	13.10 (9.05 to 17.15)
a16	2	4.83 (4.38 to 5.27)	-2.33 (-3.87 to -0.79)	24.80 (22.03 to 27.57)
a17	2	1.73 (1.09 to 2.37)	-0.51 (-2.00 to 0.99)	11.39 (9.29 to 13.49)
a18	4	9.78 (6.87 to 12.50)	-3.01 (-4.29 to -0.41)	26.18 (20.88 to 32.44)
a19	2	2.93 (2.21 to 3.66)	2.32 (1.24 to 3.40)	16.79 (12.41 to 21.16)
a20	2	3.46 (3.15 to 3.78)	1.04 (-1.21 to 3.30)	15.56 (11.67 to 19.46)
a21	3	4.40 (3.95 to 4.89)	-2.17 (-3.03 to -0.62)	16.21 (12.94 to 18.56)
a22	2	2.02 (1.27 to 2.78)	-0.75 (-2.72 to 1.22)	13.63 (9.55 to 17.71)
a23	2	2.74 (2.27 to 3.22)	0.19 (-0.93 to 1.31)	11.20 (10.05 to 12.35)
a24	2	2.73 (2.32–3.15)	0.10 (-1.09 to 1.29)	12.45 (11.64 to 13.25)
a25	2	2.30 (2.25 to 2.36)	0.52 (-0.48 to 1.51)	13.24 (12.51 to 13.98)
**Correlation coefficient (-)**	**-0.28 (-0.55 to 0.05)**	**-0.79 (-0.89 to -0.62)**	**0.59 (0.32 to 0.76)**	**-0.82 (-0.90 to -0.67)**

Based on the statistical results, it can be stated that the number of input signals does not affect the final quality of extraction, but the quality of input aECG signals plays an important role, especially in terms of noise presence and the ratio between the maternal and fetal components.

**Fig 16:**
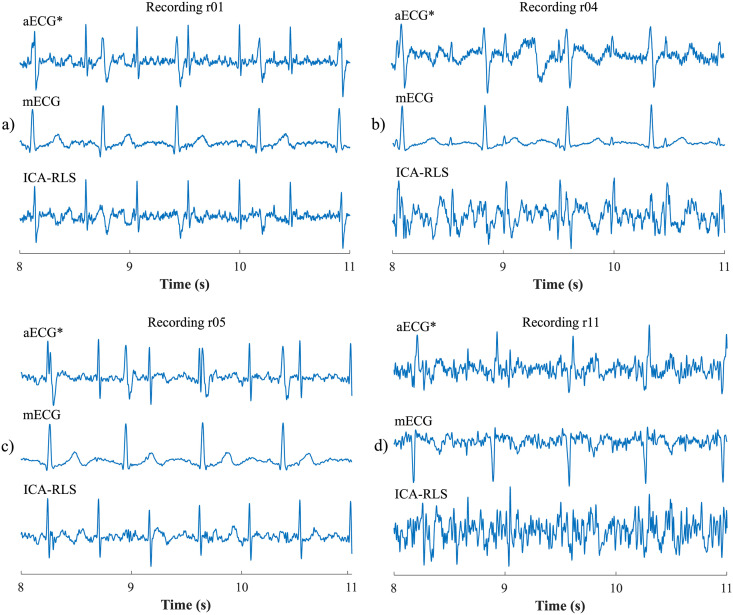
The influence of the mECG and aECG* components determined with the help of the ICA on the resulting quality of the fECG signal extracted using the ICA-RLS method. The recordings: a) r01 and c) r05 are examples of well extracted mECG and aECG* components. The recording b) r04 and d) r11 represent a less accurate extraction of the fECG. This is a result of the low magnitude of the fetal component and the presence of noise and residues of the fetal component in the mECG signal.

For the RLS algorithm, the quality of the mECG and aECG* components extracted using the ICA method is crucial. These components, forming the reference and primary inputs to the RLS system, and also the output fECG signal extracted using the ICA-RLS method, are shown in Fig 16. Examples of well-extracted fECG signals are presented for the recordings: a) r01 and c) r05 from the FECGDARHA database. It can be seen that if the ratio between the maternal and fetal components is sufficient, the algorithm is able to extract a high quality fECG. However, if the fetal component is of insufficient magnitude, and, in addition, the mECG component estimated using the ICA method contains fECG residues or noise, then the extraction by means of the RLS algorithm is less accurate, see example b) for recording r04 and d) for recording r11.

**Fig 17:**
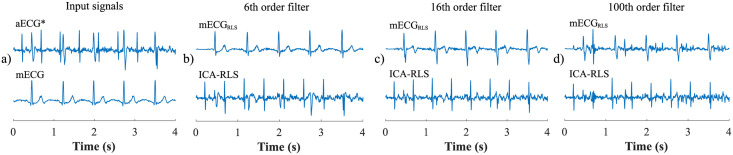
Influence of the filter order setting on the quality of the extracted fECG signal: a) aECG* and mECG signals used as the inputs to the RLS algorithm; b) low filter order settings, c) optimal filter order settings d) too high filter order settings.

With the RLS algorithm, it is very important to choose the appropriate filter order so that the input mECG signal resembles as closely as possible the mECG component in the aECG* signal. It is then possible for the RLS method to subtract the modified mECG_RLS_ component from the aECG* signal to obtain the fECG with the suppressed maternal component. The influence of the optimal filter order on the RLS algorithm is presented in Fig 17. Example a) shows the aECG* and mECG signals that were used as inputs to the algorithm in recording r05. Example b) demonstrates too low filter setting (sixth order), which led to an insufficient adjustment of the mECG component to the desired shape in the aECG* signal. The resulting fECG thus contained unnecessarily significant residues of the parent component. Example c) shows the ideal setting (16th order filter), which was able to sufficiently suppress the maternal component. Example d) shows a too high filter order (100th order filter), which was able to suppress the parent component. In addition, the fECG component was also suppressed, which affected the resulting fHR trace.

**Fig 18:**
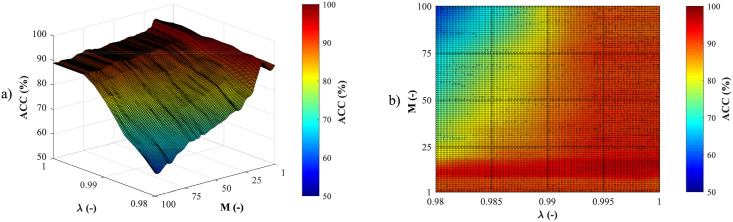
Illustration of the influence of parameter settings (filter order *M* and forgetting factor λ) in the RLS method on the resulting quality of the extracted signal a) using the front view 3D graph and b) top-down view.

Moreover, for the same record, we demonstrate the effect of different RLS-based filter setting on the quality of the extraction using a 3D graph, see Fig 18. This graph takes into account the effect of the filter order *M* and the forgetting factor λ on the resulting accuracy of detection of fQRS complexes according to the ACC parameter. The working area of the algorithm, corresponding to the red area in the graph, was defined as a system setting in which the filter is stable and achieves sufficient accuracy (ACC > 90%). It can be noted that for the record r05, the working area of the algorithm lies in the interval {*M* ∈ (1;17) ∩ λ ∈ (0.98;1)} ∪ {*M* ∈ (1;100) ∩ λ ∈ (0.994;1)}, where the global maximum (i.e. the optimal system setting) was reached for the ICA-RLS method with setting *M* = 16 and λ = 1 (ACC = 95.93%). Setting the system by values outside the working area led to a deterioration in accuracy in the detection of fQRS complexes (for example, at *M* = 100 and λ = 0.98, the resulting ACC = 58.86%). Therefore, although the input signals for this recording were of a high quality, improper setting of the RLS filter caused poor outcomes.

**Fig 19:**
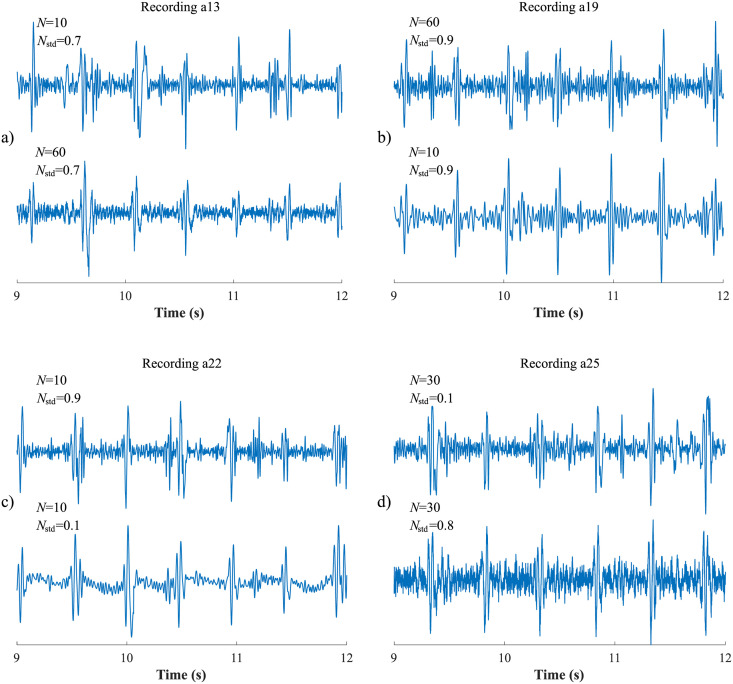
The influence of the EEMD parameters (*N* and *N*_*std*_) on the resulting quality of the fECG signal extraction. Examples a) and b) present the influence of the parameter *N*, while keeping a constant value of *N*_*std*_. Examples c) and d) present the influence of the parameter *N*_*std*_, while keeping the *N* unchanged.

When using the EEMD method, the optimal setting of the *N* and *N*_*std*_ parameters is crucial. The influence of these parameters on the resulting extraction quality is shown in Fig 19. The upper signal in all examples is the result of the most unsuccessful setting of the EEMD algorithm, while the lower signal represents the setting of the EEMD algorithm for which the best extraction results and most accurate fHR determination were achieved. Examples a) and b) represent the influence of the optimal setting of parameter *N*, while keeping *N*_*std*_ constant. If 10 ensemble trials were used for the recording a13, then the EEMD method failed to suppress the residues of the maternal component and produced worse results (ACC < 71%) comparing to *N* = 60 ensemble trials (ACC > 97%). When using 60 ensemble trials the method was able to filter the residues, and consequently, it was possible to detect the fQRS complexes more accurately. The same influence can be seen for the recording a19. However, in this case worse results were obtained using 60 ensemble trials (ACC < 75%), while better were achieved for *N* = 10 (ACC > 98%). Examples c) and d) present the influence of the optimal setting of *N*_*std*_, while keeping the same value of *N*. For the recording a22, the EEMD method failed to suppress some maternal components (ACC < 47%) when assuming *N*_*std*_ = 0.9. The use of *N*_*std*_ = 0.1 for the added noise series was more appropriate in this case, leading to the highest level of the extraction accuracy (ACC = 100%). On the contrary, for the recording a25, setting *N*_*std*_ = 0.1 produced worse results (ACC <86%), while the best extraction accuracy (ACC = 100%) was achieved using *N*_*std*_ = 0.8.

**Fig 20:**
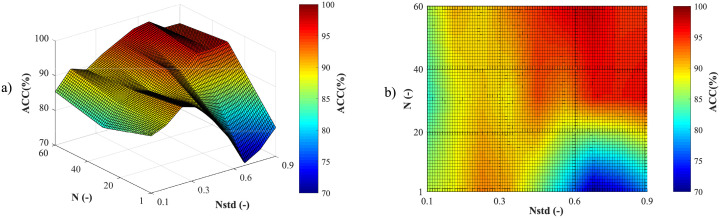
Illustration of the influence of parameter settings (*N* and *N*_*std*_) for the EEMD method on the resulting quality of the extracted signal a) using the front view of the 3D graph and b) the top-down view.

Fig 20 provides illustration of the effect of the EEMD-based filter settings (*N* and *N*_*std*_) on the resulting accuracy of the fQRS detection according to the ACC for the case of record a13. The working area of the algorithm, corresponding to the red area in the graph, lies in the interval {*N* ∈ (26;60) ∩ *N*_*std*_ ∈ (0.4;0.9)}, while the global maximum was reached for the ICA-RLS-EEMD method at the setting *N* = 60 a *N*_*std*_ = 0.7 (corresponding to ACC = 97.64%). Setting the algorithm outside the working area led to a deterioration in accuracy in the detection of fQRS complexes (for example, when *N* = 10 a *N*_*std*_ = 0.7, ACC = 70.56% was achieved).

**Fig 21:**
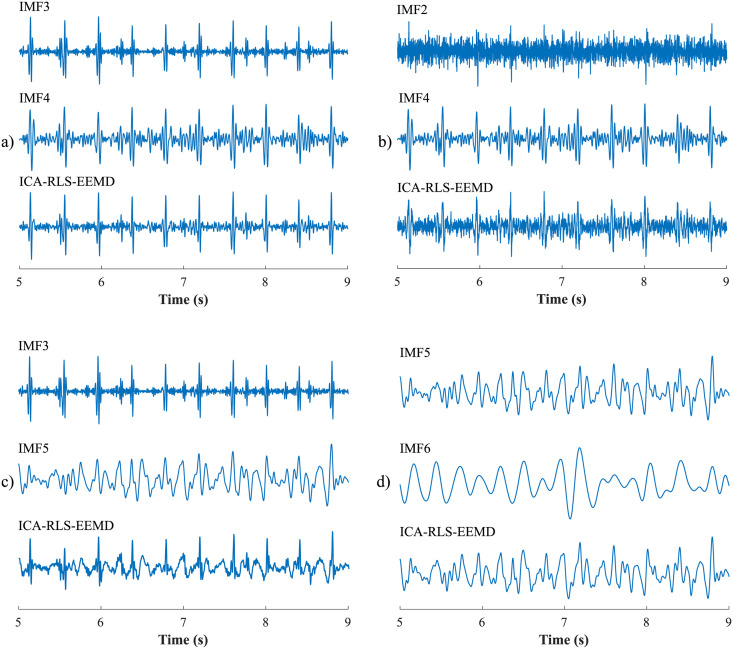
Influence of the IMF selection on the final fECG signal quality: a) the optimal selection of IMFs, b) inappropriate selection of IMF2 component representing noise, c) inappropriate selection of both IMFs leading to insufficient suppression of mECG residues, d) inappropriate selection of IMFs, where both signals contain very little information on fECG signal.

In addition to the optimal setting of the *N* and *N*_*std*_ parameters, the correct selection of extracted IMFs plays an important role since they are used to compose the resulting fECG signal. An example of the optimal selection of IMFs (IMF3 + IMF4) for r02 recording, in which ACC = 100% was achieved in the detection of fQRS complexes, is shown in Fig 21a). Example Fig 21b) presents an inappropriate selection of IMFs (IMF2 + IMF4), where the selected component IMF2 corresponds to the noise. This noise contained in the resulting signal led to an impaired detection of fQRS complexes (ACC < 92%). Example c) represents an inappropriate selection of IMFs (IMF3 + IMF5) in terms of insufficient removal of mECG residues, which also affected the accuracy of R wave detection (ACC < 94%). The last example d) demonstrates the effect of inappropriate selection of IMFs (IMF5 + IMF6), where both IMFs do not contain a well-extracted useful signal and there is a complete loss of information about the fECG signal. It is almost not recognizable even visually and accurate detection of fQRS complexes is nearly impossible (ACC < 45%).

Another important factor besides the quality of the aECG recordings is the filter setting. Improper setting of the methods either led to insufficient suppression of the interference or, on the other hand, the interference was effectively removed, however, a part of the useful signal was also filtered out along with it. Nevertheless, the functionality of the method and finding the optimal setting were demonstrated only in physiological records. It would be appropriate to verify the effectiveness of the method also on pathological records. Unfortunately, there are currently very few publicly available databases with real records that contain annotations with reference positions of fQRS complexes, and no dataset with pathological fECG records exists. Future research should therefore focus on creating a comprehensive database containing records from clinical practice. The ideal database should contain hundreds of well-scanned multichannel aECG records with a reference scalp fECG record, annotations indicating the positions of fQRS complexes, and annotations with reference T/QRS ratios. The database should contain records that have been obtained from both normal and pathological fetuses of different gestational age and different maternal health conditions. The database should include a description of how the signals were captured, information on electrode placement, gestational age of the fetus, fetal position, maternal age, maternal health, genetic predisposition, information on whether the pregnancy is at risk and whether it is single or multiple pregnancy. Such a database would be used for further research on extraction methods, which could be compared with each other both in terms of fHR determination and for deeper morphological analysis.

As we tried to obtain the most accurate fECG extraction in order to perform a morphological analysis, a relatively wide range of parameters was tested (11 combinations of aECG signals, 50 values of RLS filter order, nine values of *N*_*std*_ parameter, three values of *N* parameter, and 250 combinations of IMFs). In order to simplify the search for optimal parameters and reduce the number of algorithm runs, the number of parameters could be decreased. Such range of parameters could be chosen, for which we know that the method works relatively correctly and is sufficient for a reliable determination of fHR (e.g. four combinations of aECG signals, nine values of the RLS filter order, two values of the *N*_*std*_ parameter, one value of the *N* parameter, and 12 combinations of IMFs would be tested, see Table 11).

**Table 11:** Selection of the optimal parameters of the method tested.

Input/algorithm	Parameter	Optimal settings
aECG	Combination of electrodes	1, 4
		2, 4
		1, 3, 4
		1, 2, 3, 4
rLS	Filter order	2, 16, 32, 42, 50, 64, 86, 94, 100
EEMD	*N*	10
	*N*_*std*_	0.1, 0.9
	IMFs	3, 4, 5, 2+4, 3+4, 2+3, 2+5, 3+5, 4+5, 2+3+4, 2+3+5, 2+4+5

Reducing the computational time of the algorithm can be achieved by optimizing the hyperparameters using a shorter section of the signal and applying this setting to the entire signal. A similar approach can then be used in clinical practice. Therefore, we performed the testing on signals from the FECGDARHA database using three variants, where a signal section of 15, 30, and 60 seconds was used for optimization. Subsequently, the whole five minute recording was filtered using the method with these set of parameters and a statistical evaluation of the accuracy of detection of fQRS complexes for the whole recording was performed, see Table 12. A signal section of 15 and 30 seconds cannot be considered sufficient, as poor results were achieved in the statistical evaluation of the whole record. Optimization using a 60-second signal proved to be the most suitable and accurate variant, as the subsequent statistical evaluation of the detection of fQRS complexes for the whole recording achieved almost as accurate results as in the case of parameter optimization for the whole recording length (five minutes). According to ACC, for eight records (r01, r02, r03, r05, r06, r08, r09 and r10), the accuracy difference was less than 1%. For recording r07 the value of ACC differed by 1.17%, for recording r11 by 2.63%, for recording r12 by 2.24% and the most significant difference was for recording r04, where the value of ACC differed by 5.56%.

**Table 12:** Statistical evaluation of the detection of fQRS complexes for the whole recording, while optimizing the method only on the signal section with a length of 15, 30 and 60 seconds (the 95% confidence interval is reported in parenthesis).

**Rec.**	**15 seconds used for optimization**			
	**ACC (%)**	**SE (%)**	**PPV (%)**	**F1-score (%)**
r01	97.98 (96.58–98.92)	98.14 (96.77–99.03)	99.84 (99.12–100.00)	98.98 (98.27–99.46)
r02	96.54 (94.86–97.80)	97.27 (95.72–98.38)	99.23 (98.21–99.75)	98.24 (97.37–98.88)
r03	98.69 (97.52–99.40)	98.98 (97.90–99.59)	99.71 (98.94–99.96)	99.34 (98.75–99.70)
r04	73.88 (70.44–77.12)	81.01 (77.73–84.00)	89.35 (86.54–91.76)	84.98 (82.84–86.95)
r05	96.77 (95.10–97.99)	97.52 (96.00–98.58)	99.21 (98.17–99.74)	98.36 (97.50–98.98)
r06	91.13 (88.77–93.13)	94.51 (92.51–96.11)	96.22 (94.48–97.54)	95.36 (94.09–96.42)
r07	76.06 (73.21–80.33)	83.09 (79.16–86.59)	89.98 (86.72–91.65)	86.40 (84.03–88.47)
r08	99.69 98.90–99.96)	99.85 (99.15–100.00)	99.85 (99.15–100.00)	99.85 (99.45–99.98)
r09	96.39 (94.67–97.67)	97.41 (95.89–98.49)	98.92 (97.78–99.56)	98.16 (97.27–98.82)
r10	92.53 (90.27–94.40)	97.17 (95.57–98.32)	95.08 (93.13–96.61)	96.12 (94.91–97.11)
r11	39.65 36.44–42.92)	50.78 (47.02–54.53)	64.39 (60.25–68.37)	56.78 (53.99–59.54)
r12	79.35 (76.18–82.28)	81.90 (78.81–84.71)	96.23 (94.34–97.62)	88.49 (86.60–90.19)
**Rec.**	**30 seconds used for optimization**			
	**ACC (%)**	**SE (%)**	**PPV (%)**	**F1-score (%)**
r01	97.98 (96.58–98.92)	98.14 (96.77–99.03)	99.84 (99.12–100.00)	98.98 (98.27–99.46)
r02	99.55 (98.68–99.91)	99.70 (98.91–99.96)	99.85 (99.16–100.00)	99.77 (99.34–99.95)
r03	96.68 (95.06–97.88)	97.95 (96.59–98.88)	98.68 (97.50–99.39)	98.31 (97.48–98.93)
r04	68.83 (65.24–72.27)	75.48 (71.93–78.78)	88.66 (85.67–91.22)	81.54 (79.19–83.72)
r05	99.69 (98.88–99.96)	99.69 (98.88–99.96)	100.00 (99.43–100.00)	99.85 (99.44–99.98)
r06	87.59 (84.92–89.94)	91.10 (88.69–93.14)	95.79 (93.93–97.21)	93.38 (91.90–94.67)
r07	48.20 (44.64–51.78)	59.81 (55.85–63.67)	71.29 (67.22–75.12)	65.05 (62.22–67.80)
r08	99.69 (98.90–99.96)	99.85 (99.15–100.00)	99.85 (99.15–100.00)	99.85 (99.45–99.98)
r09	96.39 94.67–97.67)	97.41 (95.89–98.49)	98.92 (97.78–99.56)	98.16 (97.27–98.82)
r10	91.94 (89.61–93.89)	96.70 (95.00–97.95)	94.92 (92.93–96.47)	95.80 (94.56–96.83)
r11	48.60 (45.27–51.93)	61.42 (57.71–65.03)	69.95 (66.17–73.54)	65.41 (62.78–67.97)
r12	87.41 (84.74–89.77)	90.22 (87.75–92.34)	96.56 (94.84–97.83)	93.28 (91.80–94.57)
**Rec.**	**60 seconds used for optimization**			
	**ACC (%)**	**SE (%)**	**PPV (%)**	**F1-score (%)**
r01	99.69 (98.88–99.96)	99.85 (99.14–100.00)	99.85 (99.14–100.00)	99.85 (99.44–99.98)
r02	99.70 (98.91–99.96)	99.85 (99.16–100.00)	99.85 (99.16–100.00)	99.85 (99.45–99.98)
r03	98.40 (97.15–99.20)	98.68 (97.52–99.40)	99.71 (98.94–99.96)	99.19 (98.56–99.60)
r04	82.13 (79.03–84.94)	87.98 (85.18–90.41)	92.51 (90.11–94.49)	90.19 (88.39–91.79)
r05	99.69 (98.88–99.96)	99.69 (98.88–99.96)	100.00 (99.43–100.00)	99.85 (99.44–99.98)
r06	92.25 (90.01–94.13)	95.40 (93.53–96.85)	96.55 (94.86–97.80)	95.97 (94.77–96.96)
r07	90.60 (88.09–92.73)	93.78 (91.59–95.54)	96.39 (94.59–97.73)	95.07 (93.71–96.21)
r08	99.69 (98.90–99.96)	99.85 (99.15–100.00)	99.85 (99.15–100.00)	99.85 (99.45–99.98)
r09	98.94 (97.82–99.57)	99.09 (98.02–99.66)	99.85 (99.15–100.00)	99.47 (98.90–99.78)
r10	94.16 (92.10–95.82)	98.74 (97.54–99.46)	95.30 (93.40–96.79)	96.99 (95.91–97.85)
r11	50.00 (46.66–53.34)	63.12 (59.44–66.69)	70.64 (66.91–74.17)	66.67 (64.07–69.19)
r12	91.77 (89.49–93.69)	94.45 (92.46–96.04)	97.00 (95.41–98.16)	95.71 (94.49–96.73)

Another variant, the advantage of which is to maintain the efficiency of filtration in the case of fetal or maternal movement, is optimization at regular intervals. The filter would be optimized for 60 seconds, following 60 seconds would be then filtered with these optimized parameters, followed by further optimization lasting 60 seconds. In clinical practice, parameter optimization based on fHR evaluation would also be beneficial. The parameters would be again first optimized for 60 seconds and with these set of parameter values the signal would be filtered. The algorithm would monitor whether the fHR is in the expected interval. If not, the optimization would be performed again. Of course, a combination of both mentioned approaches is also possible, i.e. optimization at regular intervals and optimization based on the deviation of fHR from the expected interval.

This approach also solves the problem that arises in practical use, when we do not have annotations with reference positions of fQRS complexes. The algorithm would receive both mHR information (which can be easily obtained from the aECG record) and fHR information. Based on the information on the mR-mR positions and the estimated fR-fR, the algorithm would check whether the fHR is in the expected interval or whether it has deviated and optimization is needed. A limitation of this approach is its use in pathological records, where fHR and mHR may vary.

The problem of the absence of information on the position of fetal heart beats can be further solved by combining NI-fECG with fetal phonocardiograph. It is possible to detect fS1 fetal heart sounds in the fetal phonocardiographic signal, which correlate with R-peaks with a slight time delay.

Based on the results presented, one can notice that the ICA-RLS-EEMD method was able to extract the fECG better and to determine the fHR more accurately than the conventional ICA method as well as all the approaches presented in our previous studies. Inaccurate extraction was the result of processing aECG recordings that were not well recorded and thus contained the fetal component of too low magnitude. Therefore, in the case of clinical application, it is essential to obtain high quality aECG signals. The extraction results can be negatively influenced, in particular, by insufficient adhesion of the measuring electrodes and their placement, fetal position and maternal movements. If the aECG signals contain fetal components with a sufficient magnitude, and do not contain a large amount of noise, it will be possible to perform a highly accurate determination of a non-invasive fHR. In addition, very promising results were achieved when performing non-invasive ST segment analysis. However, the computational complexity and the need to individually set the parameters of the RLS and EEMD algorithms for each recording, are disadvantages of the ICA-RLS-EEMD algorithm. If uniform parameters were used for all recordings, extraction would be less efficient.

## Conclusion

This study develops our previous ideas on the ICA-RLS-EMD method introduced for extracting the NI-fECG from abdominal recordings. Promising results were observed, however the applied EMD method is burdened with the intrinsic limitation called mode mixing, significantly affecting the decomposition results. To prevent this phenomenon, an extended EEMD method was applied in this study. Our investigations focused on the possibility of improving extraction results using the ICA-RLS-EEMD procedure, based on the appropriate settings of the EEMD parameters. The comparison with the ICA-RLS-EMD and the other previously presented methods was shown. The evaluation was carried out by examining the ability to accurately detect the fQRS complexes and to determine the high-quality fHR. Two benchmark databases (FECGDARHA and PhysioNet Challenge 2013) were applied and the evaluation of the considered algorithms was performed using statistical parameters such as ACC, SE, PPV and F1-score. When testing the proposed ICA-RLS-EEMD on the FECGDARHA database an overall accuracy of over 80% was achieved for 11 out of 12 recordings, with an average value of ACC = 92.75% [95% confidence interval: 91.19–93.88%], SE = 95.09% [95% confidence interval: 93.68–96.03%], PPV = 96.36% [95% confidence interval: 95.05–97.17%], and F1-score = 95.69% [95% confidence interval: 94.83–96.35%]. For the Physionet Challenge 2013 database the overall accuracy above 80% was obtained for 17 out of 25 recordings, the average values of ACC = 78.24% [95% confidence interval: 73.44–81.85%], SE = 81.79% [95% confidence interval: 76.59–85.43%], PPV = 87.16% [95% confidence interval: 81.95–90.35%] and F1-score = 84.08% [95% confidence interval: 80.75–86.64%] were achieved. The ICA-RLS-EEMD method provided better results than the conventional ICA method and the previously presented approaches, including the ICA-RLS-EMD. In addition, the effectiveness of the method was also demonstrated for the ST segment analysis, which was highly accurate in 7 of 12 records. However, the main disadvantage of the ICA-RLS-EEMD method is its computational complexity and the need to individually set the parameters of the RLS and EEMD algorithms for each recording. Future research will include tests and optimization of the algorithm on signals captured using our proposed NI-fECG system. All relevant data (extracted signals from both used databases) are in S1 File named Extracted signals.

## Supporting information

S1 File Extracted signals by ICA-RLS-EEMD method. (ZIP)
