# Supplementary figures and images for: A novel algorithm based on ensemble empirical mode decomposition for non-invasive fetal ECG extraction
Source: PLoS One. 2021 Aug 13;16(8):e0256154. doi: 10.1371/journal.pone.0256154 (PMC8363249; doi:10.1371/journal.pone.0256154)

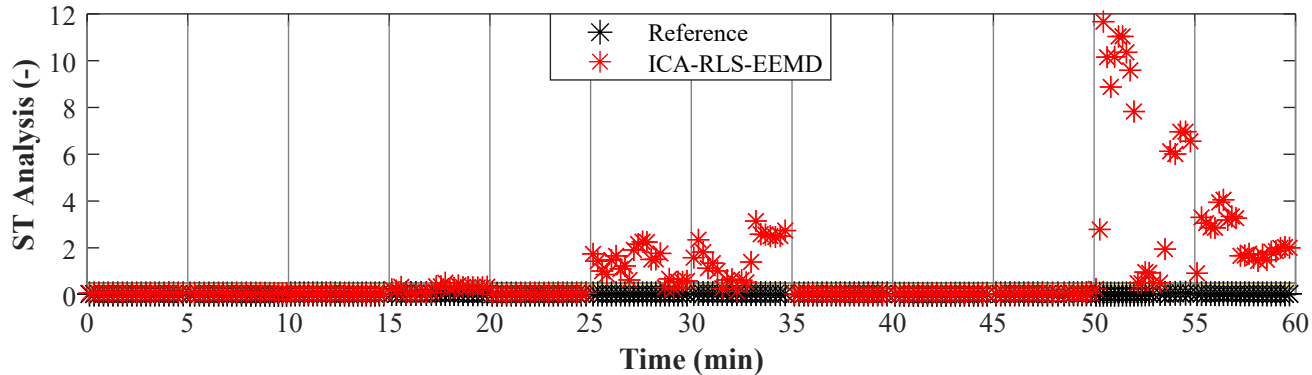

Supplement: S1 File — (ZIP) [file pone.0256154.s001.zip › Extracted signals/fig.11_ylim.pdf]
